# Levodopa Prescription Patterns in Patients with Advanced Parkinson's Disease: A Japanese Database Analysis

**DOI:** 10.1155/2023/9404207

**Published:** 2023-09-27

**Authors:** Atsushi Takeda, Toru Baba, Jun Watanabe, Masahiko Nakayama, Hiroyuki Hozawa, Miwako Ishido

**Affiliations:** ^1^Department of Neurology, National Hospital Organization Sendai Nishitaga Hospital, 2-11-11 Kagitorihoncho, Taihaku-ku, Sendai 982-8555, Japan; ^2^Department of Cognitive & Motor Aging, Tohoku University Graduate School of Medicine, 2-1 Seiryomachi, Aobaku, Sendai 980-8575, Japan; ^3^Medical, AbbVie GK, 3-1-21 Shibaura, Minato-ku, Tokyo 108-0023, Japan

## Abstract

Prescription doses of levodopa in patients with advanced Parkinson's disease (PD) are generally lower in Japan than in the United States or Europe, although Japanese guidelines for the management of PD recommend increasing the dosage as the disease progresses. However, data regarding levodopa prescription practices in patients with advanced PD in the clinical setting are limited. This retrospective observational study analyzed patterns of drug use for patients with advanced PD in Japan using claims data from hospitalized patients in the Medical Data Vision Co. database. Eligible patients had at least two PD-associated claims in two different quarters between April 1, 2008, and November 30, 2018, and a 10-item activities of daily living score <60 upon hospital discharge (as a proxy for advanced PD). The primary endpoint was the prescribed dosage of levodopa at the index hospitalization. Dosages of other PD drugs (medications with an on-label indication for PD) and non-PD drugs were also assessed. Overall, 4029 patients met the inclusion criteria (mean age, 76.9 years; 83.3% aged ≥70 years). At the index date, 74.0% were receiving levodopa. Patients received a median of one PD drug in addition to levodopa, and 27.4% and 20.2% received one or two concomitant PD drugs, respectively. Patients received a median of two non-PD drugs. The median levodopa dosage and total levodopa equivalent dosage (LED) at the index hospitalization were 418.2 and 634.8 mg/day (adjusted for body weight, 9.0 and 13.7 mg/kg/day), respectively. The median levodopa and total LED dosage in each 6-month increment during the 5 years before and after the index date ranged between 263.9 and 330.2 mg/day (5.0 and 6.5 mg/kg/day) and 402.0 and 504.9 mg/day (8.3 and 10.1 mg/kg/day), respectively. This study suggests that many Japanese patients with advanced PD could receive more intensive treatment with higher doses of levodopa.

## 1. Introduction

The global burden of Parkinson's disease (PD) has increased dramatically over the past several decades, with 6.1 million patients with PD around the world in 2016, up from 2.5 million in 1990 [1]. In Japan, the prevalence of PD has increased owing to population demographics; life expectancy in Japan is one of the longest in the world (84 years) [2], and people aged ≥65 years now account for approximately 28% of the Japanese population [3]. The most recent published studies from Japan estimate the prevalence of PD to be between 175 and 306 people per 100,000 after steady growth over the preceding 20 years [4, 5].

Patients with PD experience a range of motor and nonmotor symptoms. In addition to the cardinal motor features of rigidity, rest tremor, and bradykinesia, patients also experience nonmotor symptoms, including pain, fatigue, insomnia, depression, anxiety, apathy, psychosis, impulse control dysfunction, cognitive impairment, dementia, gastrointestinal dysfunction, autonomic dysfunction, rapid eye movement sleep behavior disorder, and olfactory dysfunction [6]. As the condition progresses, the combination of these symptoms severely affects quality of life (QoL) for these patients [7, 8]. Patients with PD experience different levels of severity in each of their disease features, creating a broad spectrum of disease that requires a tailored, individualized approach to treatment [6, 9, 10]. Data show that outcomes are improved when PD is managed by a neurologist [11]. Patient factors such as participation, choice, preference, and values may be considered during the treatment decision-making process [6, 9, 10].

The mainstay of PD treatment is dopamine replacement therapy, most often with levodopa, to manage motor symptoms [6, 9, 10]. According to the label for levodopa/carbidopa in Japan, the standard maintenance dose of levodopa/carbidopa is 600 to 750 mg/day, and the maximum dose is 1500 mg/day. Japanese guidelines recommend increasing the dose of levodopa as the disease progresses [12], but the average dosage of many drugs as prescribed in Japan is generally lower than the dosage prescribed in the United States and Europe [13, 14]. For example, a nationwide Swedish survey on levodopa use shows that more than half (54%) of patients surveyed used >400 mg of levodopa daily, with a certain proportion requiring more than 1500 mg daily [15]. According to the label in the US, the maximum dose of oral levodopa/carbidopa is 2000 mg/day for immediate-release capsules and 2450 mg/day for extended-release capsules. It is possible that Japanese physicians may be hesitant to increase the dose of levodopa because of the risk of worsening motor complications and psychological symptoms. Another potential reason for the lower average dose of levodopa in Japanese patients could be their lower body weight, given that levodopa dose/body weight has been identified as a risk factor for motor complications in several previous studies [16–19]. In addition, many drugs other than those containing levodopa are approved for the treatment of PD in Japan, either alone or concomitantly with levodopa (18 nonlevodopa drugs are described in the 2018 Japanese PD guideline [12]); as such, Japanese physicians may choose to increase the number of concomitant drugs rather than increase the dose of levodopa. However, there is limited evidence on treatment patterns in response to symptom progression in patients with advanced PD in clinical practice, and they are not well understood.

Diagnosis procedure combination (DPC) is a patient classification method developed for inpatients in the acute phase of illness in Japan and it is used in the Japanese medical service reimbursement system [20]. As of April 2020, the payment system has been applied to 1757 hospitals with a total of 483,180 beds in Japan. This number is thought to cover almost all acute inpatients and is about 30% of all hospitals with beds for general patients [20]. The DPC includes information on main diagnoses and interventions, along with patient information, such as demographic characteristics, prescriptions, diagnoses (based on the International Classification of Diseases, 10th revision (ICD-10) codes), medical treatments and procedures, test orders, and hospital admission and discharge dates. Therefore, the DPC database can be used to gather detailed statistical information on diseases.

The aim of this study was to analyze patterns of usage of levodopa and other PD drugs (drugs with an on-label indication for PD) and non-PD drugs in Japan for patients with advanced PD using the MDV database (Medical Data Vision Co., Tokyo, Japan), based on information from anonymized DPC records.

## 2. Materials and Methods

### 2.1. Database

This was a retrospective observational study using claims data from the MDV database, which records health insurance claims from acute-care hospitals that use the Japanese DPC/per-diem payment system for fixed-payment reimbursement.

All patients diagnosed with PD attending any one of the data-contributing hospitals and/or with outpatient attendance are included in the MDV database. For this analysis, the PD dataset (*N* = 174,885) comprised patients from the MDV database who had been diagnosed with PD (ICD-10 code G20) at least once. It included 380 of the 1730 DPC hospitals (21.9%) in Japan in 2018. Because records in the MDV database are anonymized, informed consent and ethics committee approval were not required, in line with the Ethical Guidelines for Epidemiological Research from the Japanese Ministry of Health, Labor, and Welfare.

### 2.2. Patients

This study evaluated claims data between April 1, 2008, and November 30, 2018. Patients with at least two claims associated with PD diagnosis (ICD-10 G20) in two different quarters and who had a 10-item activities of daily living (ADL) score <60 upon discharge from the hospital were eligible for study inclusion. The ADL score used here equates to the original Barthel index (BI), in which 10 items are each scored from 0 to 10, for a total score between 0 and 100 [21]. The BI is highly correlated with measures of disability and with QoL in patients with PD [22]. A threshold of BI < 12 has high sensitivity and specificity for detecting functional dependence (i.e., requiring nursing home care) in patients with no cognitive impairment aged ≥60 years [23] and is a threshold defining the need for assistance in daily activities (e.g., feeding, transfer, and mobility) among elderly Japanese patients [24]. An ADL score of <60 equates to a simplified BI of <12, and this score at the time of hospital discharge was used to define a patient population with advanced PD. The index date was defined as the first time at which the patient met this criterion for advanced PD (i.e., an ADL score <60 recorded on hospital discharge).

Patients were excluded if they had been diagnosed with progressive supranuclear palsy (PSP) (ICD-10 G23), secondary parkinsonism (ICD-10 G21), and parkinsonism in other diseases (ICD-10 G22) during the observation period.

### 2.3. Treatment Assessments

The primary endpoint was the prescribed dosage of PD drugs (including levodopa and any concomitant drugs) and non-PD drugs during the index hospitalization. PD drugs were defined as having an on-label indication for PD (Supplementary Table S1) and non-PD drugs as having no on-label indication but being frequently prescribed to treat PD (Supplementary Table S2). Doses of PD drugs were standardized by calculating the levodopa equivalent dose (LED) using the method reported by Tomlinson and colleagues (Supplementary Table S3) [25]. The LED serves as a practical summary of the total daily antiparkinsonian medication [25]. The secondary endpoints were the clinical and demographic characteristics of patients with advanced PD at the index date; the time course of PD drug prescriptions by analyzing the median value of prescriptions in 6-month increments before and after the index date for up to 5 years (Supplementary Figure S1); and the dose, dose/body weight escalation, treatment duration, and add-on drugs for patients with advanced PD initiated with levodopa and other PD drugs for subgroups of patients by age.

Data were collected on PD and non-PD drugs. The levodopa analysis included all forms of levodopa, with or without a decarboxylase inhibitor (DCI; i.e., levodopa, levodopa/benserazide, levodopa/carbidopa, and levodopa/carbidopa intestinal gel). Levodopa daily dose and dose/body weight before and after the index date (mg/day) were calculated at 6-month intervals, dividing the cumulative dose of levodopa in each 6-month period by 183 days.

For fixed combinations containing levodopa, only the levodopa dose was used for the LED conversion. Levodopa daily dose (or LED) at the index date was calculated by dividing the cumulative dose of levodopa (or LED) during hospitalization by the number of days of hospitalization; we also estimated the levodopa dose (or LED) by body weight using the same calculation and dividing the dose by the patient's body weight in kilograms.

### 2.4. Statistical Analysis

Continuous variables were expressed as median, interquartile range (*Q*1, *Q*3), minimum, maximum, and mean (standard deviation (SD)). Because the dataset has nonnormal distribution and large outliers, continuous variables were mainly expressed as median and interquartile range. Statistical significance was assessed using Student's *t*-test for unmatched comparisons, and the Wilcoxon signed-rank test for matched comparisons. For categorical variables, counts (frequencies) and percentages are reported. Statistical significance was assessed using the chi-squared test and Fisher's exact test for unmatched comparisons, and the McNemar test for matched comparisons. A subgroup analysis by age was conducted. The analysis software used was SAS version 9.4 or higher (SAS Institute, Cary, NC, USA).

### 2.5. Sensitivity Analysis

Because data entry errors can occur in database analyses, we conducted a sensitivity analysis to confirm the robustness of the data. In the sensitivity analysis, we excluded patients who were receiving a dosage that was higher than the maximal approved dose for the following agents: levodopa (including a DCI combination but excluding intravenous injections), duodopa, entacapone (including combinations with levodopa/carbidopa), selegiline, rasagiline, bromocriptine, cabergoline, pergolide, pramipexole, ropinirole, apomorphine, and amantadine.

## 3. Results

### 3.1. Patients

**Figure 1:**
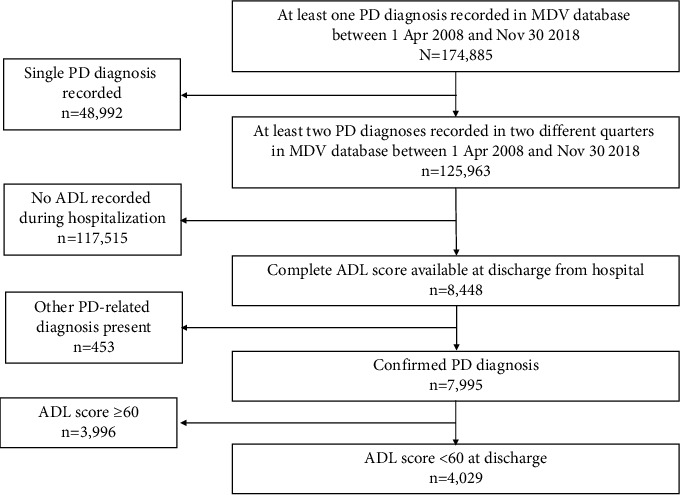
Patient disposition. ADL, activities of daily living; MDV, Medical Data Vision; PD, Parkinson's disease.

Overall, 174,885 patients from 318 facilities in the MDV database had a PD diagnosis recorded between April 1, 2008, and November 30, 2018; of these, 4029 met the eligibility criteria and were included in the analysis (Figure 1). Of the eligible patients, 2148 were female (53.3%) and 1881 were male (46.7%; Table 1). The mean (SD) age of patients was 76.9 (8.1) years, and 83.3% were aged ≥70 years.

The mean (SD) time of the preindex period and postindex follow-up for all patients was 787.7 (737.2) days and 456.2 (533.1) days, respectively. The ADL score at the index date ranged from 0 to 50 (median, 25.0). The 318 facilities providing data for this analysis were from all geographic regions of Japan, and 185 facilities with data for 3529 patients (87.6%) were on the list of medical institutions certified by the Japanese Society of Neurology (Table 2). Most patients had been admitted to hospitals with 200 to <300 beds (*n* = 998; 24.8%) or ≥500 beds (*n* = 1378; 34.2%).

### 3.2. Treatments

**Figure 2:**
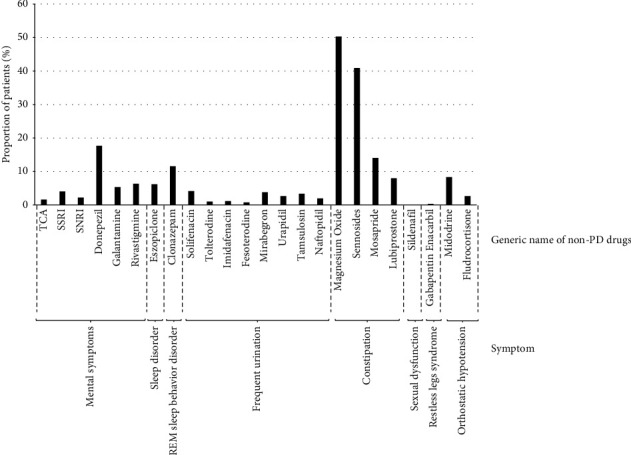
Proportion of patients receiving each type of non-Parkinson's disease medication at the index date. PD, Parkinson's disease; REM, rapid eye movement; SNRI, serotonin norepinephrine reuptake inhibitor; SSRI, selective serotonin reuptake inhibitor; TCA, tricyclic antidepressant.

**Figure 3:**
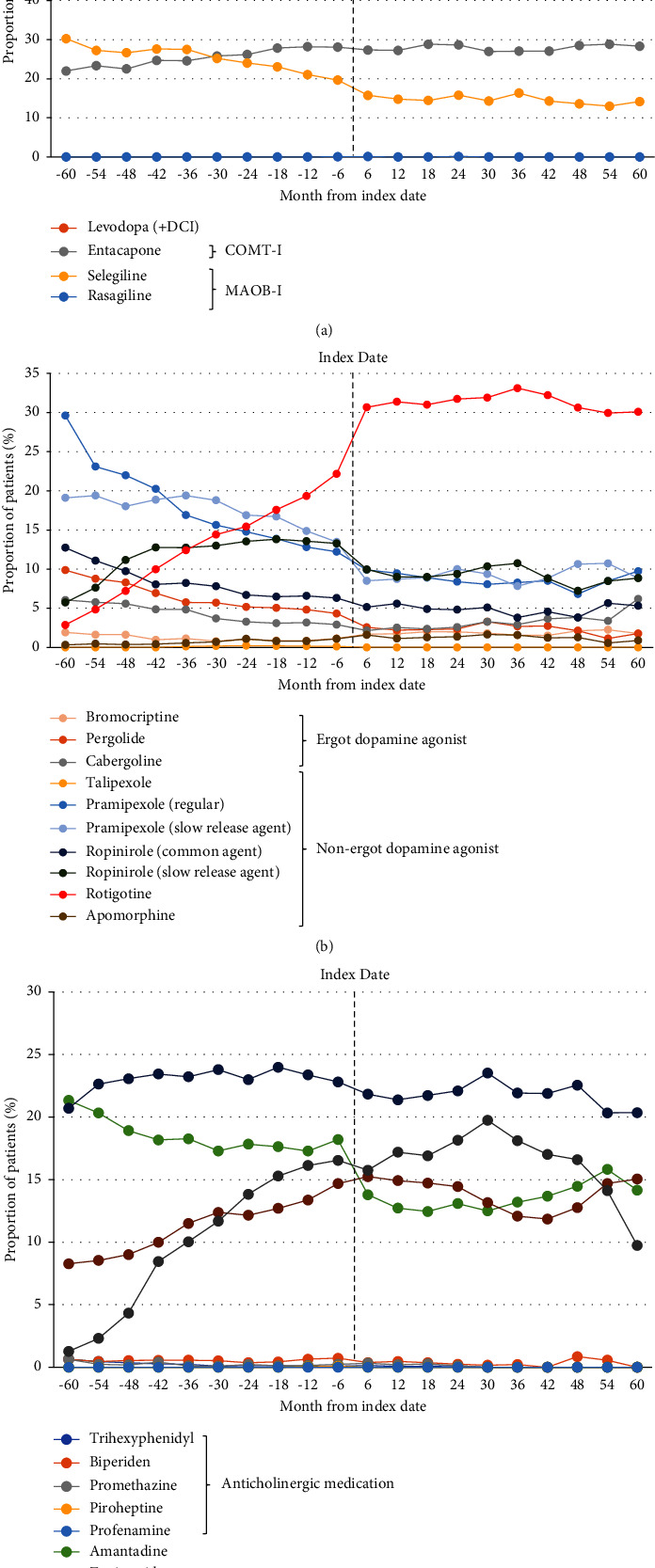
Proportion of patients receiving each type of Parkinson's disease medication in the 6-month periods before and after the index date; (a) levodopa, catechol-O-methyltransferase inhibitors (COMT-I) and monoamine oxidase B inhibitors (MAOB-I); (b) dopamine agonists; (c) anticholinergic medications and other drugs. DCI, decarboxylase inhibitor.

At the index date, 2982 patients (74.0%) were receiving levodopa, including 843 (20.9%) patients receiving entacapone (Table 3). Rotigotine was received by 952 (23.6%) patients. Patients were receiving a median of one PD drug in addition to levodopa at the index date (Table 4), but 883 patients (27.0%) were receiving no additional PD drugs, 895 (27.4%) were receiving one nonlevodopa, and 660 patients (20.2%) were receiving two nonlevodopa PD drugs. Patients also received a median of two non-PD drugs for managing nonmotor symptoms, with 527 patients (16.1%) receiving no non-PD drugs, 849 (26.0%) receiving one, and 827 (25.3%) receiving two non-PD drugs (Table 4 and Figure 2). There was a slight and gradual increase in the use of levodopa after the index date (Figure 3).

### 3.3. PD Drug Doses

**Figure 4:**
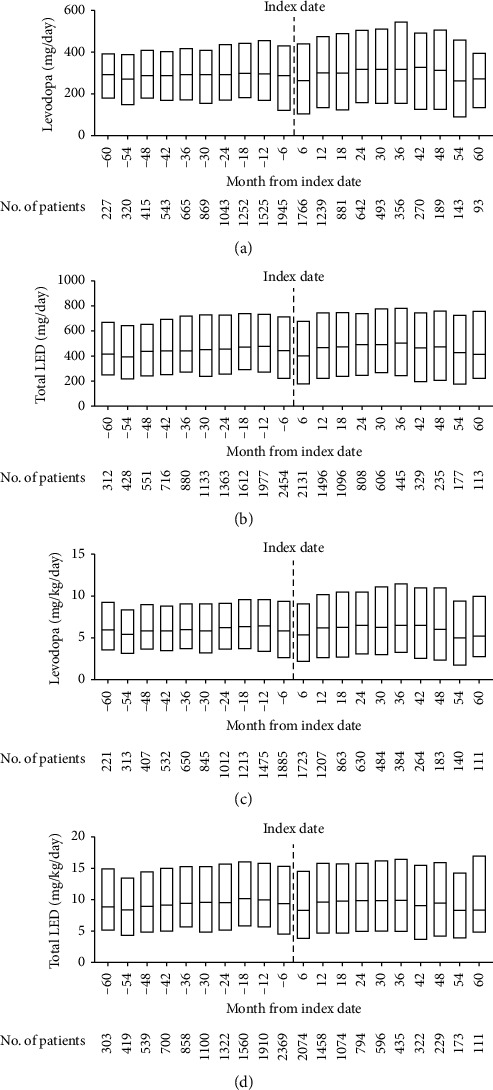
Median (*Q*1, *Q*3) levodopa dose and LED in the 6-month periods before and after the index date. (a) Levodopa daily dose; (b) daily LED; (c) levodopa body weight-adjusted dose; (d) body weight-adjusted LED. LED, levodopa equivalent dose; *Q*1/*Q*3, quartile 1/quartile 3.

At the index date, the median (*Q*1, *Q*3) levodopa ± DCI dose was 418.2 (219.2, 712.5) mg/day. The median levodopa dose in each 6-month increment for the 5 years before and after the index date varied between 263.9 and 330.2 mg/day but showed no trend toward an increase over time (Figure 4(a)). The median (*Q*1, *Q*3) LED at the index date was 634.8 (360.0, 1089.5) mg/day. The median LED in each 6-month increment for the 5 years before and after the index date varied between 402.0 and 504.9 mg/day (Figure 4(b)); similar to the levodopa dose, there was no trend toward an increase over time.

Among 3842 patients with valid body weight data available on the index date, the mean total body weight-adjusted levodopa dose and LED per day could be calculated in 2855 and 3328 patients, respectively. The mean (SD) dose and median (*Q*1, *Q*3) dose for levodopa were 14.9 (76.8) and 9.0 (4.6, 15.7) mg/kg/day, respectively, and for LED it was 22.2 (76.1) and 13.7 (7.5, 23.7) mg/kg/day. The median body weight-adjusted doses of levodopa and LED did not change markedly over time (Figures 4(c) and 4(d)), ranging from 5.0 to 6.5 mg/kg/day for levodopa and 8.3 to 10.1 mg/kg/day for LED. The mean (SD) bodyweight had increased somewhat from the index date (50.8 (11.7) kg at 5 years vs. 48.0 (11.3) kg at the index date), while body mass index (BMI) remained stable (20.9 (4.2) kg/m^2^ vs. 20.0 (4.6) kg/m^2^).

### 3.4. Subgroup Analysis by Age

There were significant differences in the male-to-female ratio between age subgroups (*p* < 0.0001), with a higher proportion of men in the younger subgroups and women in the older subgroups (age ≥60 years; Supplementary Table S4). Similarly, height, body weight, and BMI were highest in the younger age groups and significantly lower in the older subgroups (*p* < 0.0001 for height and weight; *p* = 0.0075 for BMI). The ADL scores at the index date were also significantly higher in younger versus older groups (*p* < 0.0001).

The number of PD drugs patients received at the index date also varied significantly by age (*p* < 0.0001), with younger patients tending to take more PD drugs than older patients (Supplementary Table S5). However, there was much less variation between age groups in the number of non-PD drugs (Supplementary Table S6), with the median being two non-PD drugs in all patients aged ≥50 years, and three in those aged 18 to 49 years.

The median total LED differed significantly between age groups (Supplementary Table S7). The median LED and body weight-adjusted LED were highest in those aged 50 to 59 years and decreased in older age groups (*p* < 0.0001). The median levodopa dose and the body weight-adjusted levodopa dose did not differ significantly by age.

Median doses of levodopa or LED before and after the index date did not vary markedly in any of the age groups (Supplementary Figures S2a–S2d).

### 3.5. Sensitivity Analysis

This dataset includes outlier values because the database values were used without data cleaning. A sensitivity analysis was therefore performed to investigate whether the exclusion of such outlier data would change the key results. The outlier values were not considered to have come from the group of patients who actually received a high dose but who were not classified properly in terms of dose or doses for several days were entered together as a daily dose. In addition, the data were not normally distributed, so it is difficult to statistically detect outliers. Therefore, the patients who received a dosage higher than the maximal approved dose of PD drugs were excluded. In this population, the median dose of levodopa and LED at the index date was 393.6 and 573.3 mg/day, respectively (Supplementary Table S8), compared with 418.2 and 634.8 mg/day in the overall population (Supplementary Table S7). The corresponding median weight-adjusted doses of levodopa and LED in the sensitivity analysis population were 8.4 and 12.8 mg/kg/day, respectively (Supplementary Table S9), compared with 9.0 and 13.7 mg/kg/day in the overall population (Supplementary Table S7). Median (*Q*1, *Q*3) doses and weight-adjusted doses of levodopa and LED in the 6-month increments before and after the index hospitalization are shown in Supplementary Table S10. The median and quartile values in the sensitivity analysis are consistent with those of the main analysis across all patient age groups (Supplementary Tables S8 and S9), indicating that our results are robust.

## 4. Discussion

To our knowledge, this is the first study to specifically investigate the dosage of levodopa prescribed for patients with advanced PD in Japan; previous research has not focused on this subpopulation of patients with advanced PD [26–29]. The results of our study show that the median levodopa dose in patients with advanced PD in Japan varied between 264 and 330 mg/day in the 6-month increments before and after the index date and total LED ranged from 402 to 505 mg/day. Doses were higher on the index date, reflecting the usual practice of optimizing levodopa therapy during hospitalization. Our results are consistent with previous research in Japan, which suggested that the levodopa dose prescribed by most physicians was 300 to 400 mg/day, even in patients with advanced PD [26, 30].

Several researchers have suggested that the levodopa dose used to treat PD in Japan is lower than in the United States or Europe [13, 31]. A series of international studies analyzing the efficacy and safety of different levodopa doses have used different definitions of low, medium, and high doses. The STRIDE-PD study, which was conducted across North America, Europe, and the United Kingdom, used ≤400, 400 to 600, and ≥600 mg/day to define low, medium, and high doses [32], respectively, whereas the ELLDOPA study conducted in the United States and Canada used 150, 300, and 600 mg/day [33]. According to these definitions, the patients in our study with advanced PD in Japan received low (STRIDE-PD definition) or moderate (ELLDOPA definition) doses of levodopa, which are well below the approved maximum dose. Further, in an international clinical trial of safinamide in patients starting additional therapy because of “off” time, the mean daily levodopa dose was 776 mg/day [34], whereas the mean dose in an equivalent study in Japan was between 420 and 446 mg/day [28]. In the European GLORIA registry of patients starting treatment with levodopa-carbidopa intestinal gel (LCIG), the mean LED was between 1509 and 1795 mg/day [35], whereas the mean LED for patients starting LCIG in Japan was 761 mg/day [36]. The difference in body weight between Japanese and European subjects is considered to have affected the dose, but several reports suggest that the levodopa dose is lower in Japan compared to other countries. A multicenter cross-sectional survey-based study from Korea conducted in 2007 reported that the mean levodopa dosage and LED were 487.5 mg/day and 608.9 mg/day, respectively [37]. This dosage was similar to that observed in our study, despite the focus on advanced-stage disease in this study. The body weight of Korean patients was 1.27 times higher than in our study (mean 59.6 kg for Korean patients; median 47 kg for this study). When the mean LED was plotted according to HY stage, a linear increase was observed up to stage 4 (from under 400 mg/day to over 800 mg/day), and it tended to decrease at stage 5. In our study, total LED did not change significantly over time (Figure 4). These data suggest that patients with advanced PD in Korea received an escalated dose of levodopa and agonists according to disease development. Here, our study provides real-world evidence of lower levodopa dosing in clinical practice in a large cohort of patients with PD in Japan.

Notably, 87.6% of patients in the current study were being treated at facilities with Japanese Society of Neurology certification, indicating that low levodopa doses are used even in these centers of excellence. A likely reason for this is that many patients with PD are seen by general neurologists who are not PD specialists. Overall, 30.5% of patients in our cohort were treated at centers with <300 beds; hospitals of this size may only have one specialist neurologist who may see only a small number of patients with PD per year [38]. According to a report from the Japanese Medical Specialty Board, there were 6065 board-certified neurology specialists in the Japanese Society of Neurology in September 2020 [39]. In contrast, there were only 849 members of the Movement Disorder Society of Japan in September 2020 [40], indicating that only about one in seven neurologists in Japan specializes in movement disorders. This is in line with international data indicating that treatment for most patients with PD lacks a multidisciplinary approach and effective communication between different healthcare professionals [38]. Because the magnitude of neurologist involvement is a significant predictor of outcomes, including mortality and fracture, it is essential to have a neurologist who specializes in PD leading patient management [11].

There may be several reasons why lower levodopa doses are prescribed in Japan compared with other countries. Physicians may be hesitant to increase the dose because of the risk of motor complications, which often become more frequent with a longer duration of treatment and with higher doses [41], or the development or exacerbation of neuropsychiatric symptoms [42]. Patients receiving low or reduced doses become gradually immobile, which physicians may not notice or may accept as an inevitable consequence of disease progression rather than escalating treatment to obtain a better outcome. Previous studies have shown that the benefit of an increased dose may outweigh the risks [43] and that many patients prefer mild dyskinesia to immobility [44]. Brodell and colleagues compared patients receiving levodopa at doses of ≥800 versus <800 mg/day [43]. Although there was a small and not statistically significant worsening of motor complications in the higher-dose group, this group also experienced significantly better QoL and less severe depression compared with patients receiving <800 mg/day [43]. These observations suggest that Japanese patients with advanced PD, such as those included in our study, may benefit from the escalation of levodopa dosing. Because many drugs are approved to treat PD in Japan [12], we hypothesized that Japanese physicians may choose to increase the number of concomitant drugs rather than increasing the dose of levodopa to reduce the risk of adverse effects. However, our data showed that patients received a median of only one PD drug in addition to levodopa. The Japanese Parkinson's Disease Treatment Guideline 2018 [12] recommends that physicians increase the frequency of levodopa to 4–5 times per day or add a dopamine agonist for patients who have motor fluctuations despite receiving levodopa 3 times a day. Entacapone and monoamine oxidase B inhibitors such as selegiline, rasagiline, and safinamide, as well as istradefylline or zonisamide, are recommended as add-on third-line treatments. There is no specific limit in levodopa dosage for developing motor complications. These appear mainly based on disease duration, and the heterogeneity of PD is large. In particular, PD causes impairment of intestinal absorption, which requires dose adjustment according to each patient's condition. Our data suggest that Japanese patients with advanced PD are undermedicated in terms of both the dosage of levodopa and the number of concomitant drugs.

Another potential reason for the lower dose of levodopa in Japanese patients is their lower body weight compared with patients abroad. According to the STRIDE-PD study, the dose threshold for the onset of motor complications was 4 mg/kg/day in patients with early stages of PD [32]. In Chinese patients with PD, the threshold dose was recently defined as 400 mg/day [45]. In the current study, the mean body weight of patients was 48.0 kg, whereas in the STRIDE-PD study, the mean body weight was 79.4 kg [32]. The median weight-adjusted dose of levodopa prescribed to these patients at the index date in our study was 9.0 mg/kg/day, which was above the threshold of 4 mg/kg/day for motor symptoms identified in the STRIDE-PD study. These data indicate that the levodopa dose per body weight in patients with advanced PD in Japan with severe functional impairment (ADL <60) is similar to the value of the threshold for motor complications in early-stage PD in other countries.

We defined advanced PD as an ADL score of <60 because the database did not include information about outcome measures of PD symptoms such as Hoehn & Yahr (H&Y) stages or Unified Parkinson's Disease Rating Scale (UPDRS) scores, which are generally used for the definition of advanced stages of PD [46, 47]. A consensus on indicators of suspected advanced PD and eligibility for device-aided therapies was reached by a panel of movement disorder specialists to ≥5 daily oral doses of levodopa, ≥2 hours a day of off time, or ≥1 hour a day with troublesome dyskinesia [47]. However, the database we used does not include information about the number of daily levodopa intakes, off time, nor time of troublesome dyskinesia. As reported in a previous study, BI is highly correlated with measures of disability and with QoL in patients with PD [22]; therefore, we consider ADL could be a good indicator of the progression of PD. The patients included in this study might be different from universally defined advanced PD patients. As patients in this study had a median age of 78 years and ADL dependence, a certain number of late-stage PD patients were possibly included. Coelho et al. proposed an operational definition of late-stage PD as a score on the Schwab and England Scale of less than 50% during periods of adequate symptom control (“on” period) [48]. A score of 50% corresponds with the patient requiring help with half of their chores and experiencing difficulty with all activities. Motor symptoms and NMS that are nonresponsive to levodopa are the most reliable predictors of late-stage PD [48]. Late-stage PD patients are known to adopt a lower dose of levodopa compared with advanced ones and to have a lower percentage of troublesome motor complications but a higher percentage of axial signs and dysphagia [48, 49]. It was reported that there were patients with late-stage disease who may have discontinued PD medications because of nonresponsiveness [50]. The presence of late-stage patients could be a potential reason for the low dosages of levodopa observed in this study. The results showing decreased levodopa dosage and LED at 54 and 60 months after the index date might reflect that patients had proceeded to a late stage of disease (Figure 4).

The strengths of our study are the large patient cohort and the investigation of doses as well as treatments over time. The limitations are those common to observational database analyses, namely, that we were unable to obtain detailed information on the patients' duration of PD or other factors that may affect prescribing (e.g., concomitant conditions) because the data collected in the MDV database were not verified against the patients' medical records. The lack of information about outcome measures of PD symptoms, such as H&Y stages and UPDRS scores is a limitation of this study. In addition, the MDV database is focused on major hospitals, so data from small hospitals, chronic care facilities, and clinics are underrepresented. We used the ADL score to identify patients with advanced PD, but this score was obtained in only 6.7% of hospitalized patients with PD, representing a potential for selection bias. In this study, data on patients who needed to be hospitalized was included. This could also represent a selection bias towards the inclusion of more severe patients. The differences between patients included in this study and those universally defined as advanced PD patients should be noted. A limitation of this database was that it was not possible to specify the reason for hospitalization. Because of this, patients with low ADL scores for reasons other than PD symptoms, such as falls, could be included in this study. There was limited follow-up for some patients in the cohort, as well as missing data points (e.g., whether a patient changed hospitals) and the possibility that some patients may have been counted twice if they changed from one MDV hospital to another. In addition, the proportion of patients in our cohort who were aged ≥70 years was high (83.3%), which may have biased the results. Finally, the analysis predates the 2018 Japanese PD guideline update [12] and therefore reflects prescribing practices influenced by the 2002 and 2011 guidelines [51, 52].

## 5. Conclusions

These data in a real-world population of Japanese patients with advanced PD reveal that the median levodopa dosage and total LED at the index date were 418.2 mg/day and 634.8 mg/day (adjusted for body weight, 9.0 mg/kg/day and 13.7 mg/kg/day), respectively, and that patients received a median of one PD drug in addition to levodopa. The dose of levodopa appears to be low compared with previous reports from other countries, although the difference is less marked after adjustment for body weight. Our data suggest that patients with advanced PD in Japan may benefit from an escalated dose of levodopa to manage their PD symptoms.

## Figures and Tables

**Table 1:** Patient demographic and clinical characteristics.

Characteristic	Overall population (*N* = 4029)
Sex, *n* (%)	
Male	1881 (46.7)
Female	2148 (53.3)
Age (years)	
Mean (SD)	76.9 (8.1)
Median (*Q*1, *Q*3)	78.0 (72.0, 83.0)
Age categories, *n* (%)	
18–49 years	22 (0.6)
50–59 years	96 (2.4)
60–69 years	555 (13.8)
70–79 years	1667 (41.4)
≥80 years	1689 (41.9)
Height (cm)	*n* = 3752
Mean (SD)	154.6 (10.3)
Median (*Q*1, *Q*3)	155.0 (148.0, 162.0)
Body weight (kg)	*n* = 3842
Mean (SD)	48.0 (11.3)
Median (*Q*1, *Q*3)	47.0 (39.6, 55.0)
Body mass index (kg/m^2^)	*n* = 3732
Mean (SD)	20.0 (4.6)
Median (*Q*1, *Q*3)	19.7 (17.3, 22.4)
ADL score at index date	
Mean (SD)	25.0 (21.6)
Median (*Q*1, *Q*3)	25.0 (0, 50.0)

ADL, activities of daily living; *Q*1/*Q*3, quartile 1/quartile 3; SD, standard deviation.

**Table 2:** Characteristics of participating institutes.

Participating institutions	Facilities, *n* (%)	Patients, *n* (%)
All	318 (100)	4029 (100)
Facilities with board-certified specialists from the Japanese Society of Neurology	185 (58.2)	3529 (87.6)
Board-certified facilities from the Japanese Society of Neurology	143 (45.0)	3226 (80.1)
Educational^a^	63 (19.8)	1912 (47.5)
Education associated^b^	70 (22.0)	1217 (30.2)
Education related^c^	10 (3.1)	97 (2.4)
Hospital size		
<100 beds	3 (0.9)	4 (0.1)
100–199 beds	52 (16.4)	227 (5.6)
200–299 beds	58 (18.2)	998 (24.8)
300–399 beds	78 (24.5)	715 (17.7)
400–499 beds	63 (19.8)	707 (17.5)
≥500 beds	64 (20.1)	1378 (34.2)
Region		
Hokkaido	13 (4.1)	164 (4.1)
Tohoku	27 (8.5)	214 (5.3)
Kanto	62 (19.5)	949 (23.6)
Chubu	52 (16.4)	708 (17.6)
Kinki	55 (17.3)	703 (17.4)
Shikoku	16 (5.0)	56 (1.4)
Chugoku	34 (10.7)	823 (20.4)
Kyushu and Okinawa	59 (18.6)	412 (10.2)
Hospital type		
University	15 (4.7)	289 (7.2)
Municipal	79 (24.8)	751 (18.6)
Public	101 (31.8)	1433 (35.6)
Private	123 (38.7)	1556 (38.6)

^a^Accreditation criteria for educational facilities were (i) ≥1 certified educator and ≥3 certified specialists including the educator are working full-time; (ii) ≥10 beds in the department of neurology or related departments or ≥100 hospitalized patients/year in the department of neurology or related departments; and (iii) training system and facilities available at location to enable curriculum-based training for neurologists. ^b^Accreditation criteria for education-associated facilities were (i) ≥1 full-time certified educator; (ii) ≥10 beds in the department of neurology or related departments or ≥100 hospitalized patients/year in the department of neurology or related departments; and (iii) training system and facilities available at location to enable curriculum-based training for neurologists. ^c^Accreditation criteria for education-related facilities were (i) hospital or clinic with ≥1 full-time specialist (preferably educator); (ii) cooperation with educational institutions; and (iii) training system and facilities available to enable curriculum-based training for neurologists at educational and education-associated facilities.

**Table 3:** Parkinson's disease drugs taken by patients and the median dose at the index date.

Treatment	Patients, *n* (%)	Median (*Q*1, *Q*3) daily dose	
		Unadjusted (mg LED/day)	Adjusted by body weight^a^ (mg LED/kg/day)
Any PD drug	3482 (86.4)	634.8 (360.0, 1089.5)	13.7 (7.5, 23.7)
Levodopa			
Levodopa ± DCI	2982 (74.0)	418.2 (219.2, 712.5)	9.0 (4.6, 15.7)
Entacapone^b^	843 (20.9)	680.8 (399.0, 1152.2)	14.5 (8.6, 24.2)
Ergot dopamine agonists			
Bromocriptine	65 (1.6)	65.1 (29.5, 140.1)	1.2 (0.7, 2.5)
Pergolide	86 (2.1)	60.5 (25.0, 127.2)	1.4 (0.7, 2.9)
Cabergoline	93 (2.3)	97.0 (42.8, 181.6)	2.4 (0.9, 3.7)
Nonergot dopamine agonists			
Talipexole	0 (0.0)		
Pramipexole, regular	371 (9.2)	94.7 (37.5, 204.3)	2.1 (0.8, 4.6)
Pramipexole, slow release	270 (6.7)	184.4 (66.2, 390.0)	3.7 (1.3, 7.4)
Ropinirole, regular	181 (4.5)	86.8 (26.9, 162.0)	1.7 (0.6, 3.9)
Ropinirole, slow release	304 (7.6)	117.9 (35.6, 266.7)	2.4 (0.8, 5.6)
Rotigotine	952 (23.6)	179.6 (96.4, 327.9)	3.9 (2.0, 7.4)
Apomorphine	50 (1.2)	38.8 (14.3, 133.3)	0.7 (0.3, 2.3)
Monoamine oxidase type B inhibitors			
Rasagiline	2 (0.1)	152.9 (75.9, 230.0)	3.4 (1.6, 5.1)
Selegiline	493 (12.2)	41.4 (21.9, 76.1)	0.8 (0.4, 1.7)
Anticholinergics			
Trihexyphenidyl	6 (0.2)		
Biperiden	23 (0.6)		
Promethazine	8 (0.2)		
Piroheptine	0 (0.0)		
Profenamine	1 (0.0)		
Other PD drugs			
Amantadine	540 (13.4)	103.0 (52.1, 180.1)	2.3 (1.2, 4.0)
Zonisamide	662 (16.4)		
Droxidopa	521 (12.9)		
Istradefylline	466 (11.6)		

^a^Calculated in patients who had body weight data available. ^b^Entacapone or the combination drug of entacapone + levodopa/carbidopa. DCI, decarboxylase inhibitor; LED, levodopa equivalent dose; PD, Parkinson's disease; *Q*1/*Q*3, quartile 1/quartile 3.

**Table 4:** Number of medications used concomitantly with levodopa that patients were receiving at index date.

Parameters	Patients receiving concomitant treatment	
	With PD drug^a^ (*n* = 3265)	With non-PD drug^b^ (*n* = 3265)
Number of concomitant drugs/patient		
Mean (SD)	1.8 (1.5)	2.2 (1.4)
Median (*Q*1, *Q*3)	1.0 (1.0, 3.0)	2.0 (1.0, 3.0)
Number of concomitant drugs received, *n* (%)		
0	883 (27.0)	527 (16.1)
1	895 (27.4)	849 (26.0)
2	660 (20.2)	827 (25.3)
3	437 (13.4)	578 (17.7)
4	239 (7.3)	288 (8.8)
≥5	151 (4.6)	196 (6.0)

^a^Drugs with an approved indication of PD. ^b^Drugs often used in patients with PD but not specifically indicated for PD. PD, Parkinson's disease; *Q*1/*Q*3, quartile 1/quartile 3; SD, standard deviation.

## Data Availability

The data generated and/or analyzed during the current study and used to support the findings of the study were supplied by AbbVie under license and therefore cannot be made freely available. Requests for access to these data should be made to the corresponding author.

## Supplementary Materials

Supplementary Materials Supplementary Table S1: Parkinson's disease medication used in Japan. Supplementary Table S2: non-Parkinson's disease medication. Supplementary Table S3: calculation of levodopa equivalent doses. Supplementary Table S4: demographic and clinical characteristics of patients by age group. Supplementary Table S5: number of Parkinson's disease medications used concomitantly with levodopa at the index hospitalization. Supplementary Table S6: number of non-Parkinson's disease medications used concomitantly with levodopa at the index hospitalization. Supplementary Table S7: doses of levodopa and levodopa equivalents at the index date by age subgroup. Supplementary Table S8: sensitivity analysis of Parkinson's disease drugs and doses at the index date, excluding patients receiving higher than the maximal approved dose. Supplementary Table S9: sensitivity analysis of Parkinson's disease drugs and body weight-adjusted doses at the index date, excluding patients receiving higher than the maximal approved dose. Supplementary Table S10: sensitivity analysis of median (*Q*1, *Q*3) dose of levodopa and levodopa equivalent dose, including body weight-adjusted dose, in the 6-month increments for 5 years before and after the index date, excluding patients receiving higher than the maximal approved dose. Supplementary Figure S1: study design. Supplementary Figure S2a: median (*Q*1, *Q*3) levodopa dose in the 6-month periods before and after the index date by age group. Supplementary Figure S2b: median (*Q*1, *Q*3) levodopa dose equivalent in the 6-month periods before and after the index date by age group. Supplementary Figure S2c: median (*Q*1, *Q*3) body weight-adjusted levodopa dose in the 6-month periods before and after the index date by age group. Supplementary Figure S2d: median (*Q*1, *Q*3) body weight-adjusted levodopa dose equivalents in the 6-month periods before and after the index date by age group.
