# Supplementary material for: Levodopa Prescription Patterns in Patients with Advanced Parkinson's Disease: A Japanese Database Analysis
Source: Parkinsons Dis. 2023 Sep 27;2023:9404207. doi: 10.1155/2023/9404207 (PMC10550461; doi:10.1155/2023/9404207)
Supplement: Supplementary Materials — Supplementary Table S1: Parkinson's disease medication used in Japan. Supplementary Table S2: non-Parkinson's disease medication. Supplementary Table S3: calculation of levodopa equivalent doses. Supplementary Table S4: demographic and clinical characteristics of patients by age group. Supplementary Table S5: number of Parkinson's disease medications used concomitantly with levodopa at the index hospitalization. Supplementary Table S6: number of non-Parkinson's disease medications used concomitantly with levodopa at the index hospitalization. Supplementary Table S7: doses of levodopa and levodopa equivalents at the index date by age subgroup. Supplementary Table S8: sensitivity analysis of Parkinson's disease drugs and doses at the index date, excluding patients receiving higher than the maximal approved dose. Supplementary Table S9: sensitivity analysis of Parkinson's disease drugs and body weight-adjusted doses at the index date, excluding patients receiving higher than the maximal approved dose. Supplementary Table S10: sensitivity analysis of median (Q1, Q3) dose of levodopa and levodopa equivalent dose, including body weight-adjusted dose, in the 6-month increments for 5 years before and after the index date, excluding patients receiving higher than the maximal approved dose. Supplementary Figure S1: study design. Supplementary Figure S2a: median (Q1, Q3) levodopa dose in the 6-month periods before and after the index date by age group. Supplementary Figure S2b: median (Q1, Q3) levodopa dose equivalent in the 6-month periods before and after the index date by age group. Supplementary Figure S2c: median (Q1, Q3) body weight-adjusted levodopa dose in the 6-month periods before and after the index date by age group. Supplementary Figure S2d: median (Q1, Q3) body weight-adjusted levodopa dose equivalents in the 6-month periods before and after the index date by age group. [file 9404207.f1.docx]

**Supplementary Materials**

**Supplementary Tables**

**Supplementary Table S1:** Parkinson's disease medication used in Japan.

| **Class** | **Generic name** | **Product name** |
| --- | --- | --- |
| Levodopa | Levodopa | Dopaston |
|  |  | Dopasol |
|  | Levodopa/carbidopa | Neodopaston |
|  |  | Menesit |
|  |  | Carcopa |
|  |  | Dopacol |
|  |  | Parkiston |
|  |  | Leprinton |
|  |  | Duodopa |
|  | Levodopa/benserazide | Madopar |
|  |  | EC-Doparl |
|  |  | Neo-Dopasol |
|  |  | Parlodel |
|  | Levodopa/carbidopa/ entacapone | Stalevo |
| Dopamine agonist | Bromocriptine | Parlodel |
|  |  | Bromocriptine GE |
|  | Pergolide | Permax |
|  |  | Pergolide GE |
|  | Cabergoline | Cabasar |
|  |  | Cabergoline GE |
|  | Talipexole | Domin |
|  | Pramipexole (regular) | BI-Sifrol |
|  | Pramipexole (slow-release agent) | Mirapex |
|  |  | Pramipexole (slow-release agent) GE |
|  | Pramipexole | Pramipexole GE |
|  | Ropinirole (common agent) | ReQuip |
|  |  | Ropinirole GE |
|  | Ropinirole (slow-release agent) | ReQuip CR |
|  |  | Ropinirole (slow-release agent) GE |
|  | Rotigotine | Neupro |
|  | Apomorphine | Apokyn |
| Anticholinergic medication | Trihexyphenidyl | Artane |
|  |  | Trihexyphenidyl GE |
|  | Biperiden | Akineton |
|  |  | Biperiden GE |
|  | Promethazine | Hiberna |
|  |  | Pyrethia |
|  | Piroheptine | Trimol |
|  | Profenamine | Parkin^a^ |
| Amantadine | Amantadine | Symmetrel |
|  |  | Amantadine GE |
| MAOB-I | Selegiline | FP |
|  |  | Selegiline GE |
|  | Rasagiline | Azilect |
| COMT inhibitor | Entacapone | Comtan |
|  |  | Entacapone GE |
| Zonisamide | Zonisamide | Trerief |
|  |  | Zonisamide GE |
| Droxidopa | Droxidopa | Dops |
|  |  | Droxidopa GE |
| Istradefylline | Istradefylline | Nouriast |

^a^Discontinued March 2018

COMT, catechol-O-methyltransferase; CR, controlled release; GE, generic; MAOB-I, monoamine oxidase B inhibitor

**Supplementary Table S2:** Non–Parkinson's disease medication.

| **Symptom** | **Drug name/category** | **Brand name** |
| --- | --- | --- |
| Mental symptoms | TCA |  |
|  | Clomipramine | Anafranil |
|  | Nortriptyline | Noritren |
|  | Amitriptyline | Tryptanol |
|  |  | Amitriptyl GE |
|  | Amoxapine | Amoxan |
|  | Imipramine | Tofranil |
|  |  | Imidol GE |
|  | Trimipramine | Surmontil |
|  | Lofepramine | Amplit |
|  | Dosulepin | Prothiaden |
|  | SSRI |  |
|  | Paroxetine | Paxil |
|  |  | Paxil CR |
|  |  | Paroxetine GE |
|  | Sertraline | Zoloft |
|  |  | Sertraline GE |
|  | Escitalopram | Lexapro |
|  | Fluvoxamine | Depromel |
|  |  | Luvox |
|  |  | Fluvoxamine GE |
|  | SNRI |  |
|  | Duloxetine | Cymbalta |
|  | Venlafaxine | Effexor SR |
|  | Milnacipran | Toledomin |
|  |  | Milnacipran GE |
|  | Donepezil | Aricept |
|  |  | Donepezil GE |
|  | Galantamine | Reminil |
|  | Rivastigmine | Exelon |
|  |  | Rivastach |
| Sleep disorder | Eszopiclone | Lunesta |
| REM sleep behavior disorder | Clonazepam | Rivotril |
|  |  | Landsen |
| Frequent urination | Solifenacin | Vesicare |
|  | Tolterodine | Detrusitol |
|  | Imidafenacin | Uritos |
|  |  | Staybla |
|  | Fesoterodine | Toviaz |
|  | Mirabegron | Betanis |
|  | Urapidil | Ebrantil |
|  | Tamsulosin | Harnal |
|  |  | Tamsulosin GE |
|  | Naftopidil | Flivas |
|  |  | Naftopidil GE |
| Constipation | Magnesium oxide | Magnesium oxide |
|  | Sennosides | Pursennid |
|  |  | Sennosides GE |
|  | Mosapride | Gasmotin |
|  |  | Mosapride GE |
|  | Lubiprostone | Amitiza |
| Sexual dysfunction | Sildenafil | Revatio |
| Restless legs syndrome | Gabapentin enacarbil | Gabapen |
| Orthostatic hypotension | Midodrine | Metligine |
|  |  | Midodrine GE |
|  | Fludrocortisone | Florinef |

CR, controlled release; GE, generic; REM, rapid eye movement; SNRI, serotonin norepinephrine reuptake inhibitor; SR, slow release; SSRI, selective serotonin reuptake inhibitor; TCA, tricyclic antidepressant

**Supplementary Table S3:** Calculation of levodopa equivalent doses.

| **Medication** | **Conversion factor** |
| --- | --- |
| Levodopa (DCI combination) | x1 |
| Duodopa | x1.11 |
| Entacapone (or Stalevo) | Levodopa x0.33 |
| Selegiline | x10 |
| Rasagiline | x100 |
| Bromocriptine | x10 |
| Cabergoline | x66.7 |
| Pergolide | x100 |
| Pramipexole | x100 |
| Ropinirole | x20 |
| Rotigotine | x13.3 |
| Apomorphine | x10 |
| Amantadine | x1 |

DCI, decarboxylase inhibitor

**Supplementary Table S4:** Demographic and clinical characteristics of patients by age group.

| **Characteristic** | **Overall population (N = 4,029)** | **Age group, years** | | | | |  |
| --- | --- | --- | --- | --- | --- | --- | --- |
|  |  | **18–49  (n = 22)** | **50–59  (n = 96)** | **60–69  (n = 555)** | **70–79  (n = 1667)** | **≥80  (n = 1689)** | ***P* value** |
| Sex, n (%) |  |  |  |  |  |  |  |
| Male | 1,881 (46.7) | 14 (63.6) | 55 (57.3) | 265 (47.7) | 839 (50.3) | 708 (41.9) | <0.0001 |
| Female | 2,148 (53.3) | 8 (36.4) | 41 (42.7) | 290 (52.3) | 828 (49.7) | 981 (58.1) |  |
| Age, years |  |  |  |  |  |  |  |
| Mean (SD) | 76.9 (8.1) | 43.0 (8.0) | 55.7 (2.7) | 65.7 (2.6) | 75.2 (2.8) | 84.0 (3.3) | <0.0001 |
| Median (Q1, Q3) | 78.0 (72.0, 83.0) | 45.5 (41.0, 48.0) | 56.0 (53.0, 58.0) | 66.0 (64.0, 68.0) | 75.0 (73.0, 78.0) | 80.0 (81.0, 86.0) |  |
| Height, cm | n = 3752 | n = 22 | n = 92 | n = 524 | n = 1543 | n = 1571 |  |
| Mean (SD) | 154.6 (10.3) | 164.1 (7.8) | 161.8 (9.7) | 157.9 (9.5) | 155.5 (9.8) | 152.0 (10.3) | <0.0001 |
| Median (Q1, Q3) | 155.0 (148.0, 162.0) | 164.5 (160.0, 170.0) | 162.0 (155.0, 168.5) | 158.0 (150.0, 165.0) | 155.0 (149.0, 163.0) | 151.0 (145.0, 159.0) |  |
| Body weight, kg | n = 3842 | n = 22 | n = 94 | n = 528 | n = 1586 | n = 1612 |  |
| Mean (SD) | 48.0 (11.3) | 57.4 (10.5) | 55.3 (14.6) | 51.2 (11.9) | 48.6 (11.3) | 45.7 (10.3) | <0.0001 |
| Median (Q1, Q3) | 47.0 (39.6, 55.0) | 58.4 (50.9, 66.3) | 53.3 (44.4, 65.0) | 50.1 (42.4, 57.6) | 48.0 (40.0, 56.0) | 44.6 (38.0, 52.6) |  |
| BMI, kg/m^2^ | n = 3732 | n = 22 | n = 92 | n = 520 | n = 1537 | n = 1561 |  |
| Mean (SD) | 20.0 (4.6) | 21.3 (3.8) | 21.1 (5.2) | 20.5 (3.9) | 20.0 (3.8) | 19.8 (5.4) | 0.0075 |
| Median (Q1, Q3) | 19.7 (17.3, 22.4) | 21.5 (18.9, 22.9) | 20.4 (17.4, 23.9) | 20.1 (17.6, 22.9) | 19.8 (17.3, 22.6) | 19.4 (17.1, 22.1) |  |
| ADL score at index date |  |  |  |  |  |  |  |
| Mean (SD) | 25.0 (21.6) | 36.6 (19.2) | 33.7 (22.4) | 30.3 (21.3) | 26.3 (21.5) | 21.3 (21.2) | <0.0001 |
| Median (Q1, Q3) | 25.0 (0, 50.0) | 45.0 (30.0, 50.0) | 45.0 (5.0, 50.0) | 40.0 (5.0, 50.0) | 30.0 (0, 50.0) | 15.0 (0, 45.0) |  |

ADL, activities of daily living; BMI, body mass index; Q1/Q3, quartile 1/quartile 3; SD, standard deviation

**Supplementary Table S5:** Number of Parkinson's disease medications used concomitantly with levodopa at the index hospitalization.

| **Parameter** | **All patients (N = 3265)** | **Age group, years** | | | | | ***P* value** |
| --- | --- | --- | --- | --- | --- | --- | --- |
|  |  | **18–49  (n = 16)** | **50–59  (n = 77)** | **60–69  (n = 456)** | **70–79  (n = 1349)** | **≥80  (n = 1367)** |  |
| Number of concomitant medications per patient |  |  |  |  |  |  |  |
| Mean (SD) | 1.8 (1.5) | 3.1 (1.6) | 3.0 (1.8) | 2.2 (1.6) | 1.9 (1.5) | 1.4 (1.3) | <0.0001 |
| Median (Q1, Q3) | 1.0 (1.0, 3.0) | 3.0 (2.0, 4.0) | 3.0 (2.0, 4.0) | 2.0 (1.0, 3.0) | 2.0 (1.0, 3.0) | 1.0 (0, 2.0) |  |
| Number of concomitant medications received, n (%) |  |  |  |  |  |  |  |
| 0 | 883 (27.0) | 2 (12.5) | 11 (14.3) | 77 (16.9) | 317 (23.5) | 476 (34.8) | <0.0001 |
| 1 | 895 (27.4) | 2 (12.5) | 11 (14.3) | 125 (27.4) | 339 (25.1) | 418 (30.6) | 0.0008 |
| 2 | 660 (20.2) | 3 (18.8) | 10 (13.0) | 78 (17.1) | 314 (23.3) | 255 (18.7) | 0.0044 |
| 3 | 437 (13.4) | 5 (31.3) | 14 (18.2) | 88 (19.3) | 196 (14.5) | 134 (9.8) | <0.0001 |
| 4 | 239 (7.3) | 2 (12.5) | 15 (19.5) | 50 (11.0) | 111 (8.2) | 61 (4.5) | <0.0001 |
| 5 | 89 (2.7) | 1 (6.3) | 9 (11.7) | 22 (4.8) | 40 (3.0) | 17 (1.2) | <0.0001 |
| 6 | 46 (1.4) | 0 | 7 (9.1) | 11 (2.4) | 24 (1.8) | 4 (0.3) | <0.0001 |
| 7 | 13 (0.4) | 1 (6.3) | 0 | 3 (0.7) | 7 (0.5) | 2 (0.2) | 0.0274 |
| 8 | 3 (0.1) | 0 | 0 | 2 (0.4) | 1 (0.1) | 0 | 0.0854 |

Q1/Q3, quartile 1/quartile 3; SD, standard deviation

**Supplementary Table S6:** Number of non-Parkinson's disease medications used concomitantly with levodopa at the index hospitalization.

| **Parameter** | **All patients (N = 3265)** | **Age group, years** | | | | | ***P* value** |
| --- | --- | --- | --- | --- | --- | --- | --- |
|  |  | **18–49  (n = 16)** | **50–59  (n = 77)** | **60–69  (n = 456)** | **70–79  (n = 1349)** | **≥80  (n = 1367)** |  |
| Number of concomitant medications per patient |  |  |  |  |  |  |  |
| Mean (SD) | 2.2 (1.4) | 2.6 (1.9) | 2.1 (1.6) | 2.1 (1.5) | 2.3 (1.5) | 2.1 (1.3) | 0.0060 |
| Median (Q1, Q3) | 2.0 (1.0, 3.0) | 2.5 (1.0, 4.0) | 2.0 (1.0, 3.0) | 2.0 (1.0, 3.0) | 2.0 (1.0, 3.0) | 2.0 (1.0, 3.0) |  |
| Number of concomitant medications received, n (%) |  |  |  |  |  |  |  |
| 0 | 527 (16.1) | 3 (18.8) | 14 (18.2) | 80 (17.5) | 198 (14.7) | 232 (17.0) | 0.4321 |
| 1 | 849 (26.0) | 5 (31.3) | 18 (23.4) | 127 (27.9) | 330 (24.5) | 369 (27.0) | 0.4518 |
| 2 | 827 (25.3) | 1 (6.3) | 17 (22.1) | 98 (21.5) | 347 (25.7) | 364 (26.6) | 0.0783 |
| 3 | 578 (17.7) | 2 (12.5) | 17 (22.1) | 77 (16.9) | 245 (18.2) | 237 (17.3) | 0.7655 |
| 4 | 288 (8.8) | 2 (12.5) | 8 (10.4) | 44 (9.7) | 124 (9.2) | 110 (8.1) | 0.7094 |
| 5 | 125 (3.8) | 2 (12.5) | 2 (2.6) | 18 (4.0) | 65 (4.8) | 38 (2.8) | 0.0236 |
| 6 | 46 (1.4) | 1 (6.3) | 0 | 8 (1.8) | 26 (1.9) | 11 (0.8) | 0.0343 |
| 7 | 17 (0.5) | 0 | 0 | 3 (0.7) | 9 (0.7) | 5 (0.4) | 0.6808 |
| 8 | 5 (0.2) | 0 | 0 | 1 (0.2) | 3 (0.2) | 1 (0.1) | 0.5407 |
| 9 | 2 (0.1) | 0 | 0 | 0 | 2 (0.2) | 0 | 0.4787 |
| ≥10 | 1 (0.03) | 0 | 1 (1.3) | 0 | 0 | 0 | 0.0285 |

Q1/Q3, quartile 1/quartile 3; SD, standard deviation

**Supplementary Table S7:** Doses of levodopa and levodopa equivalents at the index date by age subgroup.

| **Dose** | **All patients** | **Age group, years** | | | | | ***P* value** |
| --- | --- | --- | --- | --- | --- | --- | --- |
|  |  | **18–49** | **50–59** | **60–69** | **70–79** | **≥80** |  |
| Total LED, mg/day | n = 3482 | n = 18 | n = 85 | n = 495 | n = 1454 | n = 1430 |  |
| Mean (SD) | 1024.7 (4063.8) | 1003.8 (1053.3) | 1400.4 (2031.5) | 1101.6 (1459.4) | 1158.3 (5981.9) | 840.1 (1670.0) | 0.2462 |
| Median (Q1, Q3) | 634.8 (360, 1089.5) | 722.4  (360.7, 1368.8) | 895.0  (538.5, 1458.3) | 809.4  (463.0, 1377.2) | 671.5  (385.0, 1132.7) | 545.3  (300.0, 918.1) | < 0.0001 |
| Total body weight–adjusted LED, mg/kg/day | n = 3328 | n = 18 | n = 83 | n = 472 | n = 1390 | n = 1365 |  |
| Mean (SD) | 22.2 (76.1) | 17.5 (17.0) | 26.0 (33.6) | 22.9 (35.7) | 24.8 (109.3) | 19.2 (38.1) | 0.3924 |
| Median (Q1, Q3) | 13.7  (7.5, 23.7) | 13.7  (5.4, 20.6) | 18.5  (10.3, 27.6) | 16.3  (9.2, 27.5) | 14.2  (8.1, 24.9) | 12.5  (6.7, 21.0) | < 0.0001 |
| Levodopa dose, mg/day | n = 2982 | n = 13 | n = 68 | n = 395 | n = 1210 | n = 1296 |  |
| Mean (SD) | 690.2 (4191.0) | 336.5 (319.4) | 769.7 (1398.4) | 625.0 (1055.8) | 804.5 (6423.0) | 602.8 (1205.3) | 0.7950 |
| Median (Q1, Q3) | 418.2  (219.2, 712.5) | 143.8  (88.9, 537.8) | 351.4  (91.7, 841.3) | 417.7  (225.0, 733.9) | 435.7  (229.1, 726.3) | 409.0  (210.0, 687.8) | 0.1264 |
| Levodopa body weight–adjusted dose, mg/kg/day | n = 2855 | n = 13 | n = 67 | n = 380 | n = 1158 | n = 1237 |  |
| Mean (SD) | 14.9 (76.8) | 6.2 (6.0) | 14.6 (22.5) | 13.2 (26.9) | 16.7 (115.6) | 13.8 (29.4) | 0.8661 |
| Median (Q1, Q3) | 9.0  (4.6, 15.7) | 2.7  (1.4, 10.6) | 7.5  (2.2, 16.4) | 8.9  (4.6, 14.5) | 9.1  (4.6, 16.0) | 9.0  (4.8, 15.9) | 0.0981 |

LED, levodopa equivalent dose; Q1/Q3, quartile 1/quartile 3; SD, standard deviation

**Supplementary Table S8**: Sensitivity analysis of Parkinson's disease drugs and doses at the index date, excluding patients receiving higher than the maximal approved dose.

| **Treatment** | **All patients (N = 4,026)** | **Age group, years** | | | | |  |
| --- | --- | --- | --- | --- | --- | --- | --- |
|  |  | **18–49**  **(n = 22)** | **50–59**  **(n = 96)** | **60–69**  **(n = 555)** | **70–79**  **(n = 1666)** | **≥80**  **(n = 1687)** | ***P* value** |
| Total LED dose, mg/day | | | | | | | |
| Patients, n (%) | 2891 (71.8) | 13 (59.1) | 60 (62.5) | 374 (67.4) | 1192 (71.6) | 1252 (74.2) | 0.0029 |
| Mean (SD) | 870.6 (1,393.0) | 767.9 (652.2) | 1,401.2 (2,337.4) | 968.0 (1,448.9) | 922.4 (1,362.4) | 767.9 (1,341.0) | 0.0008 |
| Median (Q1, Q3) | 573.3  (322.1, 982.6) | 601.4  (360.7, 875.0) | 757.8  (467.1, 1385.4) | 705.1  (399.0, 1236.4) | 601.0  (348.5, 1,028.5) | 511.1  (284.5, 867.5) | <0.0001 |
| Levodopa dose, mg/day | | | | | | | |
| Patients, n (%) | 2754 (68.4) | 13 (59.1) | 61 (63.5) | 362 (65.2) | 1110 (66.6) | 1208 (71.6) | 0.0048 |
| Mean (SD) | 579.4 (1,058.5) | 336.5 (319.4) | 748.2 (1,441.2) | 569.8 (1,047.7) | 588.8 (836.9) | 567.7 (1,215.3) | 0.6444 |
| Median (Q1, Q3) | 393.6  (198.7, 674.2) | 143.8  (88.9, 537.8) | 344.0  (71.4, 802.3) | 398.2  (208.7, 700.0) | 402.0  (203.0, 685.6) | 383.3  (196.5, 646.8) | 0.3009 |
| Entacapone dose^a^, mg/day | | | | | | | |
| Patients, n (%) | 737 (18.3) | 8 (36.4) | 35 (36.5) | 148 (26.7) | 336 (20.2) | 210 (12.5) | <0.0001 |
| Mean (SD) | 889.3 (1,126.8) | 1,110.0 (1,314.4) | 910.3 (1,216.3) | 846.5 (648.3) | 927.4 (1,376.0) | 846.7 (913.5) | 0.876 |
| Median (Q1, Q3) | 638.4  (375.5, 1,097.3) | 461.1  (352.8, 1,398.5) | 631.8  (254.3, 1170.4) | 715.0  (399.0, 1,130.6) | 647.9  (379.1, 1,078.4) | 591.2  (359.1, 969.8) | 0.687 |
| Amantadine dose, mg/day | | | | | | | |
| Patients, n (%) | 505 (12.5) | 6 (27.3) | 20 (20.8) | 73 (13.2) | 229 (13.8) | 177 (10.5) | 0.0007 |
| Mean (SD) | 152.9 (303.2) | 163.8 (86.3) | 167.3 (137.1) | 163.7 (125.1) | 177.6 (424.6) | 114.4 (136.9) | 0.3415 |
| Median (Q1, Q3) | 100.0  (50.0, 170.0) | 175.4  (119.3, 220.3) | 150.5  (74.4, 202.2) | 123.3  (70.0, 233.3) | 104.6  (54.4, 175.0) | 83.3  (33.3, 137.0) | 0.0002 |
| Rotigotine dose, mg/day | | | | | | | |
| Patients, n (%) | 952 (23.7) | 5 (22.7) | 29 (30.2) | 180 (32.4) | 410 (24.6) | 328 (19.4) | <0.0001 |
| Mean (SD) | 271.2 (379.3) | 246.1 (175.1) | 222.6 (259.8) | 275.0 (313.0) | 286.6 (331.8) | 254.5 (469.1) | 0.767 |
| Median (Q1, Q3) | 179.6  (96.4, 327.9) | 210.3  (186.5, 303.5) | 176.3  (79.8, 249.5) | 180.1  (98.3, 391.6) | 192.6  (106.4, 359.1) | 164.3  (87.6, 299.3) | 0.1569 |
| Selegiline dose, mg/day | | | | | | | |
| Patients, n (%) | 445 (11.1) | 3 (13.6) | 18 (18.8) | 71 (12.8) | 216 (13.0) | 137 (8.1) | <0.0001 |
| Mean (SD) | 57.3 (66.6) | 43.4 (21.4) | 45.0 (33.8) | 49.5 (44.1) | 61.4 (81.0) | 56.7 (53.8) | 0.6376 |
| Median (Q1, Q3) | 39.0  (20.7, 71.4) | 50.0  (19.4, 60.7) | 37.7  (23.2, 58.3) | 34.6  (16.4, 72.2) | 40.3  (21.8, 71.9) | 39.5  (20.7, 75.0) | 0.9568 |
| Pramipexole (regular) dose, mg/day | | | | | | | |
| Patients, n (%) | 352 (8.7) | 2 (9.1) | 12 (12.5) | 56 (10.1) | 153 (9.2) | 129 (7.7) | 0.2059 |
| Mean (SD) | 164.5 (270.0) | 267.9 (158.6) | 164.5 (246.6) | 195.5 (256.2) | 182.4 (324.9) | 128.3 (196.7) | 0.4052 |
| Median (Q1, Q3) | 88.1  (35.4, 181.4) | 267.9  (155.8, 380.0) | 82.9  (24.5, 224.6) | 126.7  (47.1, 218.5) | 86.5  (38.7, 198.7) | 71.7  (25.0, 150.0) | 0.0393 |
| Pramipexole (slow-release) dose, mg/day | | | | | | | |
| Patients, n (%) | 235 (5.8) | 5 (22.7) | 9 (9.4) | 55 (9.9) | 105 (6.3) | 61 (3.6) | <0.0001 |
| Mean (SD) | 257.7 (322.3) | 236.5 (122.3) | 173.9 (221.7) | 278.4 (384.9) | 288.2 (328.1) | 200.5 (267.1) | 0.4477 |
| Median (Q1, Q3) | 150.0  (62.5, 323.1) | 311.5  (131.3, 321.4) | 42.0  (24.2, 225.0) | 210.4  (69.7, 358.3) | 170.3  (73.1, 377.9) | 131.3  (37.5, 237.5) | 0.0908 |
| Ropinirole (slow-release) dose, mg/day | | | | | | | |
| Patients, n (%) | 289 (7.2) | 2 (9.1) | 18 (18.8) | 53 (9.6) | 133 (8.0) | 83 (4.9) | <0.0001 |
| Mean (SD) | 246.4 (645.1) | 30.0 (7.9) | 427.0 (1019.3) | 309.4 (977.4) | 230.6 (594.4) | 197.6 (240.7) | 0.6127 |
| Median (Q1, Q3) | 113.6  (34.3, 260.0) | 30.0  (24.4, 35.6) | 196.7  (59.1, 298.7) | 145.5  (41.0, 265.0) | 83.9  (33.9, 240.0) | 112.0  (31.7, 273.9) | 0.2489 |
| Ropinirole (regular) dose, mg/day | | | | | | | |
| Patients, n (%) | 165 (4.1) | 0 | 3 (3.1) | 18 (3.2) | 88 (5.3) | 56 (3.3) | 0.0435 |
| Mean (SD) | 120.8 (227.2) | – | 227.4 (371.5) | 103.6 (91.4) | 101.8 (105.0) | 150.5 (355.8) | – |
| Median (Q1, Q3) | 69.0  (24, 139.6) | – | 20.0  (5.9, 656.3) | 73.7  (16.3, 187.5) | 77.3  (27.2, 127.6) | 60.7  (22.7, 135.7) | – |
| Cabergoline dose, mg/day | | | | | | | |
| Patients, n (%) | 81 (2.0) | 1 (4.6) | 6 (6.3) | 13 (2.3) | 42 (2.5) | 19 (1.1) | 0.0008 |
| Mean (SD) | 115.7 (132.9) | 26.5 | 228.5 (118.3) | 135.5 (81.0) | 103.5 (139.2) | 98.3 (143.1) | 0.22 |
| Median (Q1, Q3) | 84.3  (35.1, 140.1) | 26.5 | 259.4  (209.0, 300.2) | 118.0  (66.7, 198.1) | 78.4  (26.9, 125.7) | 73.0  (16.4, 106.1) | 0.029 |

^a^Entacapone or the combination of entacapone + levodopa/carbidopa (Stalevo)

LED, levodopa equivalent dose; Q1/Q3, quartile 1/quartile 3; SD, standard deviation

**Supplementary Table S9**: Sensitivity analysis of Parkinson's disease drugs and body weight–adjusted doses at the index date, excluding patients receiving higher than the maximal approved dose.

| **Treatment** | **All patients (N = 3,840)** | **Age group, years** | | | | | ***P* value** |
| --- | --- | --- | --- | --- | --- | --- | --- |
|  |  | **18–49  (n = 22)** | **50–59  (n = 94)** | **60–69  (n = 528)** | **70–79  (n = 1585)** | **≥80  (n = 1611)** |  |
| Total LED dose, mg/kg/day | | | | | | | |
| Patients, n (%) | 2759 (71.9) | 13 (59.1) | 58 (61.7) | 357 (67.6) | 1135 (71.6) | 1196 (74.2) | 0.0032 |
| Mean (SD) | 19.2 (33.4) | 13.5 (11.4) | 25.7 (38.1) | 20.5 (37.4) | 20.3 (32.9) | 17.5 (32.5) | 0.1169 |
| Median (Q1, Q3) | 12.8 (7.0, 21.8) | 12.3 (5.4, 16.3) | 14.6 (10.0, 24.6) | 14.4 (7.4, 24.2) | 13.2 (7.3, 23.2) | 11.7 (6.5, 20.0) | 0.0002 |
| Levodopa dose, mg/kg/day | | | | | | | |
| Patients, n (%) | 2635 (68.6) | 13 (59.1) | 60 (63.8) | 350 (66.3) | 1060 (66.9) | 1152 (71.5) | 0.0194 |
| Mean (SD) | 12.8 (25.2) | 6.2 (6.0) | 14.2 (22.7) | 12.2 (27.1) | 12.7 (18.6) | 13.1 (29.7) | 0.8338 |
| Median (Q1, Q3) | 8.4 (4.2, 14.8) | 2.7 (1.4, 10.6) | 7.5 (1.0, 16.0) | 8.1 (3.9, 14.0) | 8.5 (4.2, 15.0) | 8.5 (4.4, 15.0) | 0.1491 |
| Entacapone dose^a^, mg/kg/day | | | | | | | |
| Patients, n (%) | 704 (18.3) | 8 (36.4) | 35 (37.2) | 139 (26.3) | 324 (20.4) | 198 (12.3) | <0.0001 |
| Mean (SD) | 19.4 (27.5) | 18.1 (19.9) | 16.3 (21.3) | 17.1 (13.2) | 20.9 (34.9) | 19.2 (21.9) | 0.6722 |
| Median (Q1, Q3) | 13.8 (7.9, 22.3) | 8.7 (5.6, 24.6) | 10.7 (5.6, 20.4) | 14.3 (8.4, 23.6) | 14.1 (8.4, 22.8) | 13.4 (7.6, 21.7) | 0.5632 |
| Amantadine dose, mg/kg/day | | | | | | | |
| Patients, n (%) | 473 (12.3) | 6 (27.3) | 19 (20.2) | 69 (13.1) | 213 (13.4) | 166 (10.3) | 0.0012 |
| Mean (SD) | 3.4 (6.2) | 3.1 (2.1) | 3.0 (2.2) | 3.5 (2.7) | 4.0 (8.7) | 2.6 (2.8) | 0.3334 |
| Median (Q1, Q3) | 2.2 (1.1, 3.8) | 2.9 (2.3, 3.3) | 2.6 (1.7, 4.0) | 3.0 (1.5, 5.2) | 2.2 (1.2, 3.9) | 2.1 (0.9, 3.2) | 0.056 |
| Rotigotine dose, mg/kg/day | | | | | | | |
| Patients, n (%) | 917 (23.9) | 5 (22.7) | 29 (30.9) | 173 (32.8) | 398 (25.1) | 312 (19.4) | <0.0001 |
| Mean (SD) | 5.9 (7.7) | 5.0 (3.8) | 4.1 (4.4) | 5.6 (6.0) | 6.4 (7.3) | 5.7 (9.3) | 0.4421 |
| Median (Q1, Q3) | 3.9 (2.0, 7.4) | 4.1 (3.1, 7.7) | 3.0 (1.4, 4.8) | 3.9 (2.0, 8.4) | 4.1 (2.3, 7.8) | 3.6 (1.8, 6.9) | 0.1112 |
| Selegiline dose, mg/kg/day | | | | | | | |
| Patients, n (%) | 427 (11.1) | 3 (13.6) | 17 (18.1) | 69 (13.1) | 205 (12.9) | 133 (8.3) | < 0.0001 |
| Mean (SD) | 1.2 (1.3) | 0.8 (0.5) | 0.9 (0.8) | 1.0 (1.0) | 1.3 (1.6) | 1.2 (1.2) | 0.4281 |
| Median (Q1, Q3) | 0.8 (0.4, 1.5) | 0.8 (0.3, 1.2) | 0.7 (0.3, 1.0) | 0.7 (0.4, 1.3) | 0.8 (0.4, 1.5) | 0.8 (0.4, 1.7) | 0.4603 |
| Pramipexole (regular) dose, mg/kg/day | | | | | | | |
| Patients, n (%) | 335 (8.7) | 2 (9.1) | 11 (11.7) | 52 (9.9) | 146 (9.2) | 124 (7.7) | 0.3431 |
| Mean (SD) | 3.6 (6.4) | 4.0 (2.0) | 1.6 (1.4) | 4.3 (6.1) | 4.1 (7.9) | 3.0 (4.6) | 0.4802 |
| Median (Q1, Q3) | 1.9 (0.7, 4.3) | 4.0 (2.6, 5.4) | 1.2 (0.3, 2.8) | 2.7 (1.1, 4.6) | 2.0 (0.8, 4.7) | 1.6 (0.6, 3.7) | 0.0816 |
| Pramipexole (slow-release) dose, mg/day | | | | | | | |
| Patients, n (%) | 226 (5.9) | 5 (22.7) | 9 (9.6) | 53 (10.0) | 100 (6.3) | 59 (3.7) | <0.0001 |
| Mean (SD) | 5.2 (6.4) | 4.8 (2.9) | 3.7 (5.0) | 5.3 (6.3) | 5.6 (6.1) | 4.7 (7.3) | 0.846 |
| Median (Q1, Q3) | 3.3 (1.2, 6.3) | 5.1 (2.7, 6.1) | 1.2 (0.4, 5.1) | 3.7 (1.3, 7.6) | 3.7 (1.5, 6.6) | 2.9 (0.7, 5.3) | 0.2088 |
| Ropinirole, slow-release dose, mg/kg/day | | | | | | | |
| Patients, n (%) | 279 (7.3) | 2 (9.1) | 17 (18.1) | 52 (9.9) | 129 (8.1) | 79 (4.9) | <0.0001 |
| Mean (SD) | 5.4 (15.9) | 0.5 (0.1) | 8.2 (19.4) | 7.3 (25.9) | 5.0 (14.7) | 4.3 (5.0) | 0.752 |
| Median (Q1, Q3) | 2.3 (0.8, 5.3) | 0.5 (0.4, 0.5) | 3.7 (1.4, 6.0) | 2.6 (0.8, 5.1) | 2.0 (0.8, 4.8) | 2.8 (0.9, 5.9) | 0.3796 |
| Ropinirole (regular) dose, mg/kg/day | | | | | | | |
| Patients, n (%) | 161 (4.2) | 0 | 3 (3.2) | 17 (3.2) | 85 (5.4) | 56 (3.5) | 0.0621 |
| Mean (SD) | 2.9 (6.1) | – | 4.9 (7.8) | 2.4 (2.2) | 2.4 (2.9) | 3.7 (9.6) | – |
| Median (Q1, Q3) | 1.5 (0.5, 3.2) | – | 0.5 (0.2, 13.8) | 1.7 (0.3, 4.0) | 1.7 (0.6, 3.0) | 1.4 (0.5, 3.1) | – |
| Cabergoline dose, mg/kg/day |  |  |  |  |  |  |  |
| Patients, n (%) | 78 (2.0) | 1 (4.6) | 5 (5.3) | 12 (2.3) | 42 (2.7) | 18 (1.1) | 0.0016 |
| Mean (SD) | 2.6 (3.1) | 0.5 | 4.3 (2.4) | 3.0 (1.8) | 2.4 (3.5) | 2.4 (3.0) | 0.655 |
| Median (Q1, Q3) | 1.7 (0.7, 3.3) | 0.5 | 4.9 (4.9, 5.7) | 3.0 (1.4, 4.2) | 1.7 (0.5, 2.9) | 1.6 (0.4, 3.0) | 0.0937 |

^a^Entacapone or the combination of entacapone + levodopa/carbidopa (Stalevo).

LED, levodopa equivalent dose; Q1/Q3, quartile 1/quartile 3; SD, standard deviation

**Supplementary Table S10:** Sensitivity analysis of median (Q1, Q3) dose of levodopa and levodopa equivalent dose, including body weight–adjusted dose, in the 6-month increments for 5 years before and after the index date, excluding patients receiving higher than the maximal approved dose.

| **Six-month increment** | **Levodopa dose** | | | | **LED** | | | |
| --- | --- | --- | --- | --- | --- | --- | --- | --- |
|  | **n** | **Median (Q1, Q3) unadjusted dose, mg/day** | **n** | **Median (Q1, Q3) weight–adjusted dose, mg/kg/day** | **n** | **Median (Q1, Q3) unadjusted dose, mg/day** | **n** | **Median (Q1, Q3) weight–adjusted dose, mg/kg/day** |
| –60 to –54 months | 209 | 295.1 (183.6, 411.2) | 203 | 6.0 (3.7, 9.4) | 249 | 399.2 (248.6, 612.7) | 240 | 8.7 (5.2, 13.9) |
| –54 to –48 months | 288 | 272.1 (147.5, 381.8) | 281 | 5.4 (3.1, 8.5) | 342 | 367.9 (196.7, 577.1) | 333 | 8.0 (3.9, 13.0) |
| –48 to –42 months | 372 | 289.6 (168.3, 403.1) | 366 | 5.8 (3.6, 8.9) | 437 | 426.2 (219.4, 639.3) | 427 | 8.7 (4.5, 14.2) |
| –42 to –36 months | 488 | 287.7 (172.1, 395.9) | 479 | 5.8 (3.4, 8.9) | 566 | 410.0 (229.5, 658.6) | 552 | 8.8 (4.8, 14.2) |
| –36 to –30 months | 598 | 295.9 (180.3, 419.7) | 584 | 6.0 (3.7, 9.2) | 704 | 417.7 (248.3, 671.8) | 685 | 8.9 (5.2, 14.5) |
| –30 to –24 months | 776 | 291.4 (147.7, 414.2) | 754 | 5.8 (3.1, 9.2) | 894 | 431.1 (218.6, 655.2) | 866 | 9.2 (4.4, 14.7) |
| –24 to –18 months | 928 | 294.5 (165.0, 433.9) | 900 | 6.1 (3.5, 9.1) | 1088 | 419.6 (239.2, 674.2) | 1052 | 9.0 (4.8, 14.5) |
| –18 to –12 months | 1110 | 298.4 (172.7, 442.6) | 1074 | 6.3 (3.5, 9.5) | 1294 | 447.3 (273.2, 684.4) | 1249 | 9.3 (5.3, 14.9) |
| –12 to –6 months | 1359 | 295.1 (155.7, 443.7) | 1313 | 6.3 (3.2, 9.4) | 1593 | 446.5 (237.7, 687.0) | 1534 | 9.3 (4.9, 14.8) |
| –6 months to index | 1731 | 275.4 (113.1, 413.1) | 1676 | 5.5 (2.3, 9.1) | 1981 | 411.2 (196.7, 651.6) | 1907 | 8.7 (4.0, 14.3) |
| Index to +6 months | 1594 | 252.5 (98.4, 428.4) | 1554 | 5.2 (2.0, 8.8) | 1715 | 371.0 (164.1, 640.1) | 1667 | 7.6 (3.4, 13.6) |
| +6 to +12 months | 1114 | 291.8 (119.7, 459.0) | 1084 | 5.9 (2.3, 9.8) | 1191 | 431.2 (203.5, 696.5) | 1159 | 8.7 (4.1, 14.6) |
| +12 to +18 months | 799 | 295.1 (110.9, 478.1) | 783 | 6.1 (2.3, 10.0) | 878 | 436.1 (199.2, 700.0) | 858 | 9.0 (4.2, 14.5) |
| +18 to +24 months | 586 | 309.8 (147.5, 491.8) | 575 | 6.2 (2.9, 10.3) | 647 | 461.9 (221.1, 702.1) | 634 | 9.1 (4.4, 14.5) |
| +24 to +30 months | 450 | 310.7 (153.0, 505.5) | 442 | 6.1 (2.7, 11.0) | 487 | 458.9 (234.4, 708.4) | 478 | 9.4 (4.7, 15.5) |
| +30 to +36 months | 326 | 320.5 (158.5, 531.2) | 319 | 6.5 (3.2, 11.5) | 358 | 496.2 (243.4, 747.5) | 349 | 9.6 (5.0, 15.3) |
| +36 to +42 months | 256 | 330.6 (126.2, 498.2) | 251 | 6.5 (2.5, 11.1) | 273 | 447.5 (155.9, 721.3) | 267 | 8.4 (3.4, 14.8) |
| +42 to +48 months | 179 | 315.9 (109.8, 497.3) | 174 | 6.1 (2.1, 11.0) | 196 | 437.4 (185.4, 701.0) | 191 | 8.4 (3.4, 13.7) |
| +48 to +54 months | 136 | 255.5 (88.0, 445.2) | 133 | 5.0 (1.7, 9.0) | 146 | 383.5 (145.8, 650.0) | 142 | 7.6 (2.8, 12.5) |
| +54 to +60 months | 87 | 265.6 (104.4, 390.2) | 86 | 5.2 (2.5, 8.4) | 90 | 350.5 (196.2, 674.9) | 88 | 7.8 (3.9, 13.8) |

LED, levodopa equivalent dose; Q1/Q3, quartile 1/quartile 3

**Supplementary Figures**

**
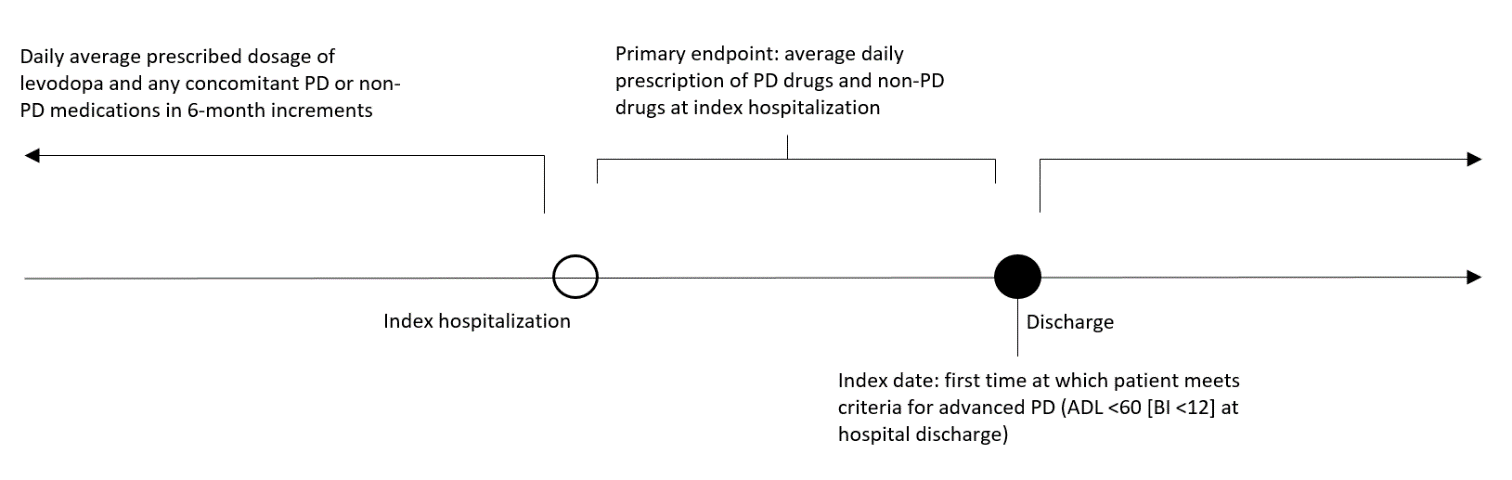
Supplementary Figure S1:** Study design. ADL, activities of daily living; BI, Barthel Index; PD, Parkinson’s disease.

**Supplementary Figure S2a:** Median (Q1, Q3) levodopa dose in the 6-month periods before and after the index date by age group. Q1/Q3, quartile 1/quartile 3.


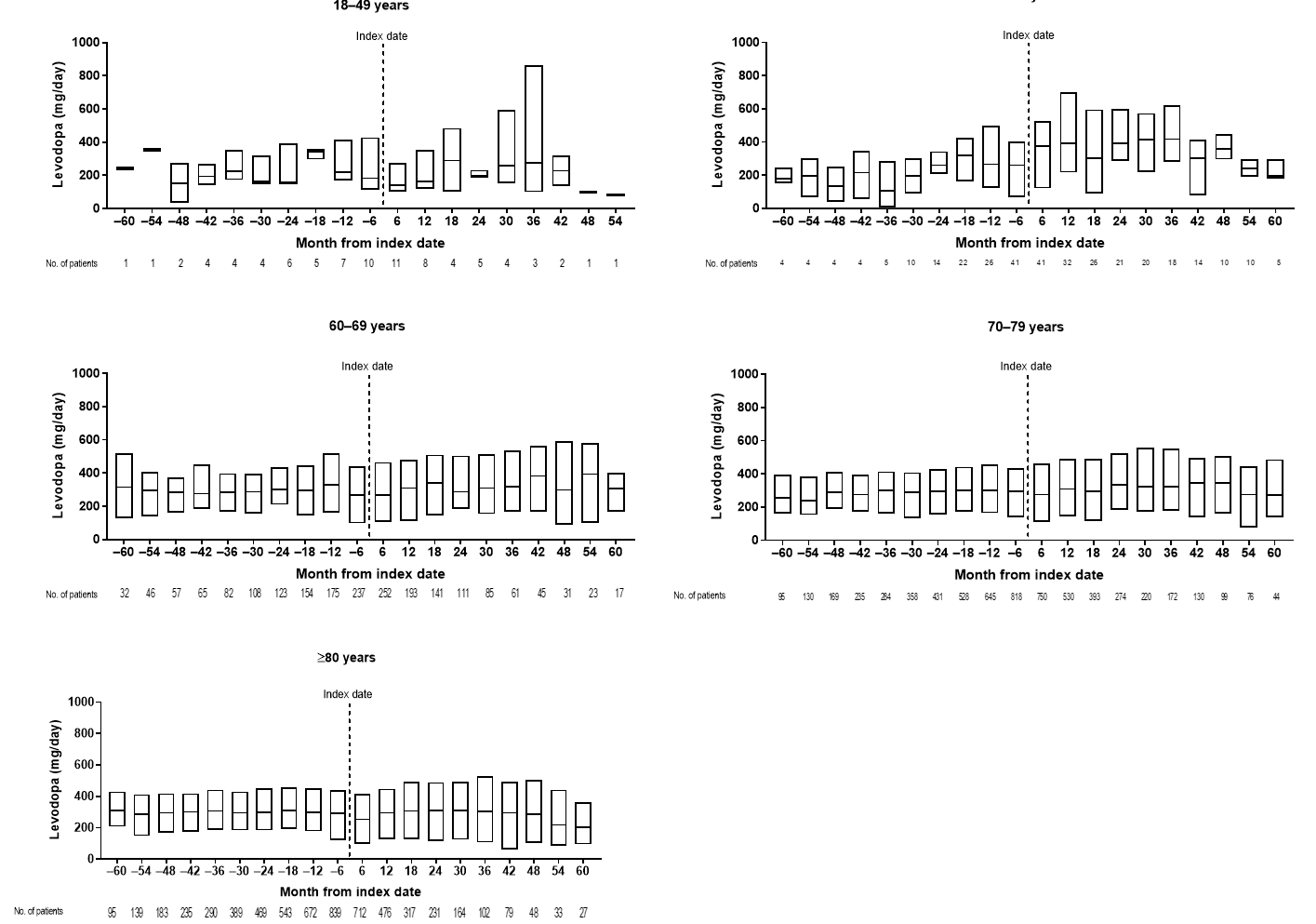


**50-59 years**


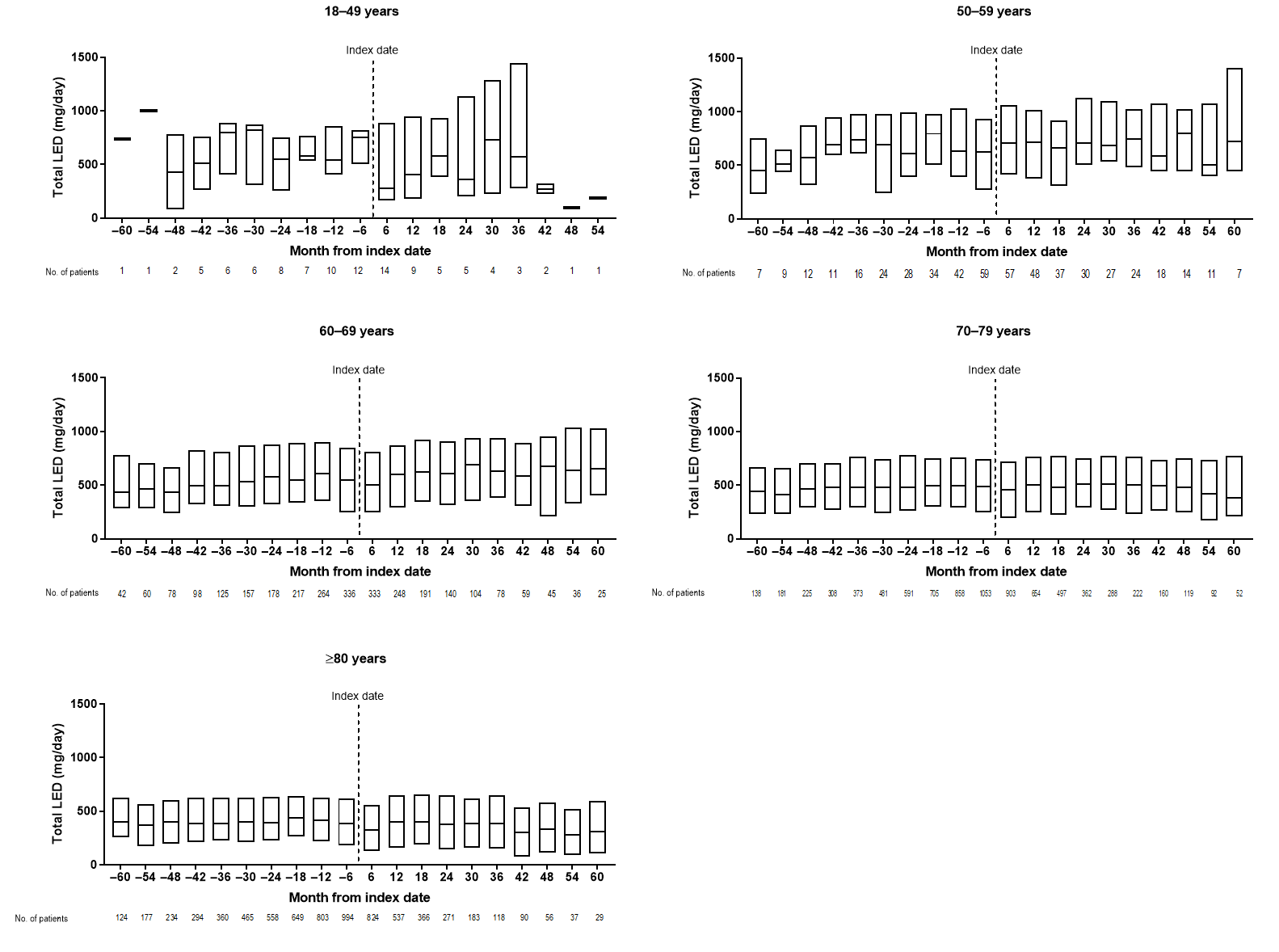
**Supplementary Figure S2b:** Median (Q1, Q3) levodopa dose equivalent in the 6-month periods before and after the index date by age group. LED, levodopa equivalent dose; Q1/Q3, quartile 1/quartile 3.


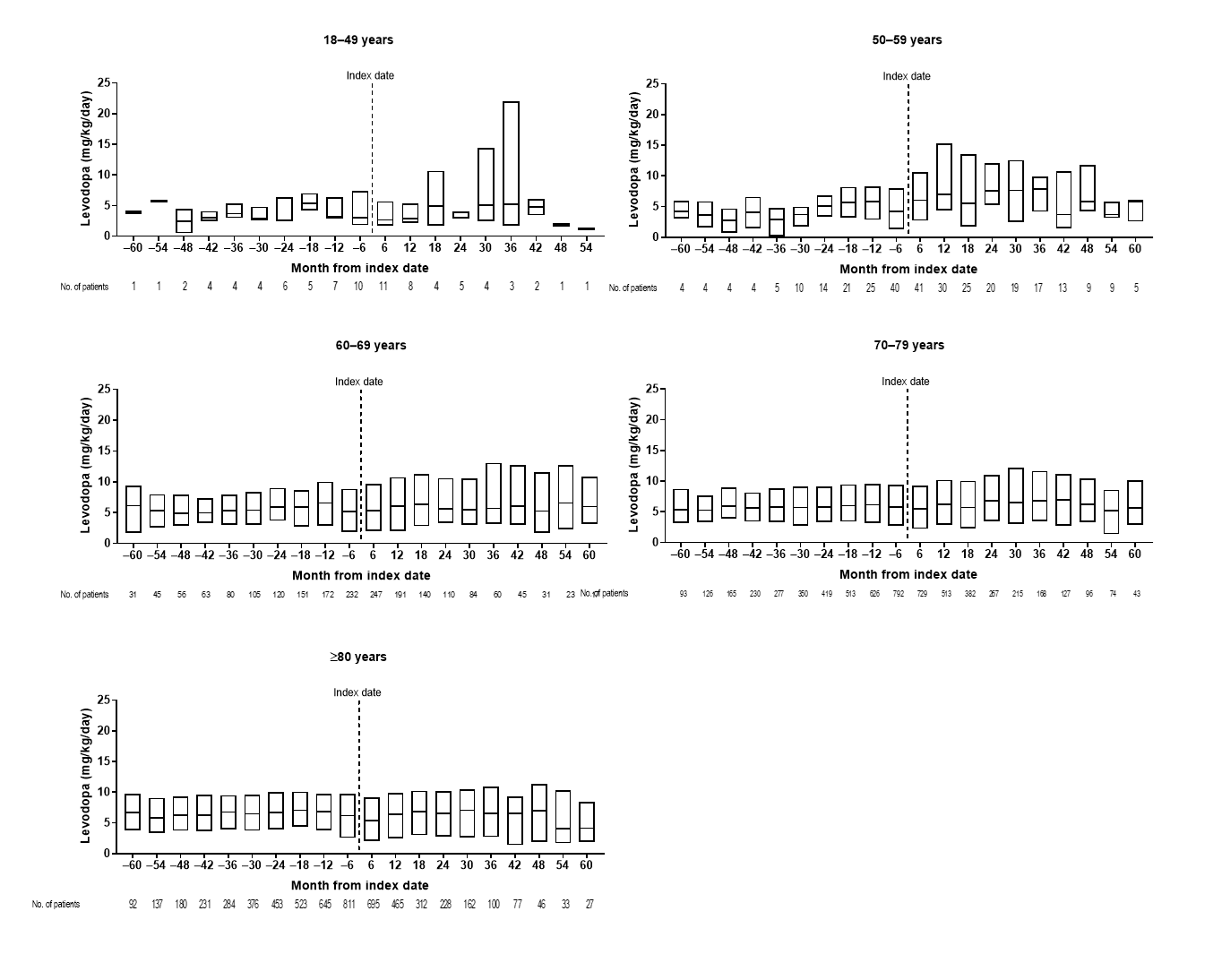
**Supplementary Figure S2c**: Median (Q1, Q3) body weight–adjusted levodopa dose in the 6-month periods before and after the index date by age group. Q1/Q3, quartile 1/quartile 3.


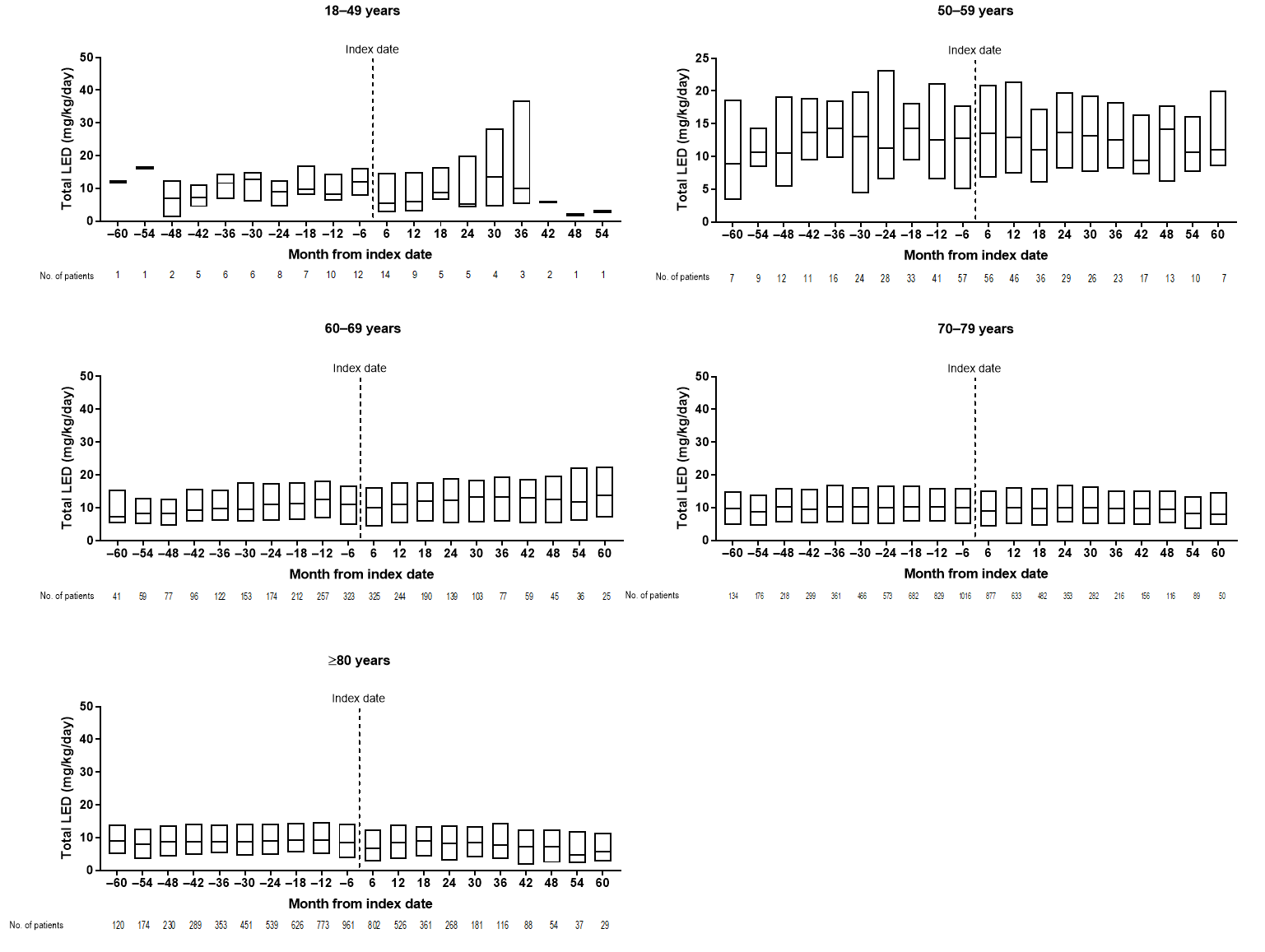
**Supplementary Figure S2d:** Median (Q1, Q3) body weight–adjusted levodopa dose equivalents in the 6-month periods before and after the index date by age group. LED, levodopa equivalent dose; Q1/Q3, quartile 1/quartile 3.
